# Lifestyle Modifications Prior to Pregnancy and Their Impact on Maternal and Perinatal Outcomes: A Review

**DOI:** 10.3390/jcm14186582

**Published:** 2025-09-18

**Authors:** Lincoln C. Kartchner, Addison Dunn, Kaitlyn H. Taylor, Mir M. Ali, Nirvana A. Manning, Nafisa K. Dajani, Everett F. Magann

**Affiliations:** 1 Department of Obstetrics & Gynecology, Virginia Tech Carilion School of Medicine, Roanoke, VA 24016, USA; 2 Department of Obstetrics & Gynecology, University of Arkansas for Medical Sciences, Little Rock, AR 72205, USA; 3 Institute for Digital Health and Innovation, University of Arkansas for Medical Sciences, Little Rock, AR 72205, USA

**Keywords:** preconception care, preconception counseling, pregnancy preparation

## Abstract

**Background/Objective**: The preconception period serves as a time for patients to modify behaviors, pre-existing disease, and potential perinatal risk factors. The purpose of this review is to review preconception behavioral modification and its effect on pregnancy outcomes. **Methods**: Electronic databases (PUBMED, CINAHL, and Embase) were searched. The search terms used were “healthy lifestyle” OR “life style” AND “fertility” OR “preconception care” OR “pre-pregnancy care” OR “pregnancy preparation”. Years searched were 1990–2024. Papers had to be in English. **Results**: Of the 301 abstracts identified, 189 full articles were selected to form this review. Preconception care (weight management, nutrition, management of pre-existing conditions, cessation of substance use, limitation to harmful environmental exposures, optimization of maternal mental health) has been shown to improve perinatal outcomes. While the benefit of preconception care has been established, compliance among reproductive aged patients is low. Reasons for low compliance in optimal preconception behavior appear to be multifactorial. While some programs have demonstrated promise in improving preconception care, more research needs to be done to improve counseling from providers as well as compliance among patients with the ultimate goal of optimizing perinatal outcomes. **Conclusions**: A comprehensive approach to physical, emotional, nutritional, environmental, and social well-being is essential to preconception care.

## 1. Introduction

The preconception period has been defined as a time prior to or between pregnancies. It includes the 100 days prior to conception and is a time period when lifestyle changes can be made to enhance maternal health prior to pregnancy. Any underlying medical conditions and health concerns that may impact maternal and perinatal health can be treated to optimize maternal health before conception. Pre-conception interventions shown to be helpful include diet and micronutrient supplementation, physical activity, obesity/weight management, cessation of smoking/recreational drug use/alcohol, addressing mental health issues, and oral hygiene [1].

Risk factors that can influence pregnancy outcomes include genetic diseases in individuals or their families, a history of prior miscarriages, premature births, fetal growth restriction, and chronic maternal diseases (hypertension, diabetes, renal disease, autoimmune disorders) [2]. Concerns in the environment such as air pollution and presence of heavy metals are also important concerns in preconception care (PCC) [3].

The effects of individual lifestyles, prior pregnancy outcomes, genetic disorders, and chronic underlying medical conditions are known to impact conception and maternal/perinatal outcomes. Although it is well known that PCC and counseling are beneficial and protocols for intervention are available, less than half of health care providers in one study used these protocols [4]. Implementation of lifestyle modifications prior to pregnancy has the potential to transform maternal and perinatal health [5].

While the focus of this paper is on maternal lifestyle modifications and preconception counseling, it is important to note that paternal health and lifestyle modification also plays a role in pregnancy [6].

The purpose of this review is to analyze maternal healthcare preconception interventions to determine their influence on maternal and perinatal health.

## 2. Methods

**Figure 1:**
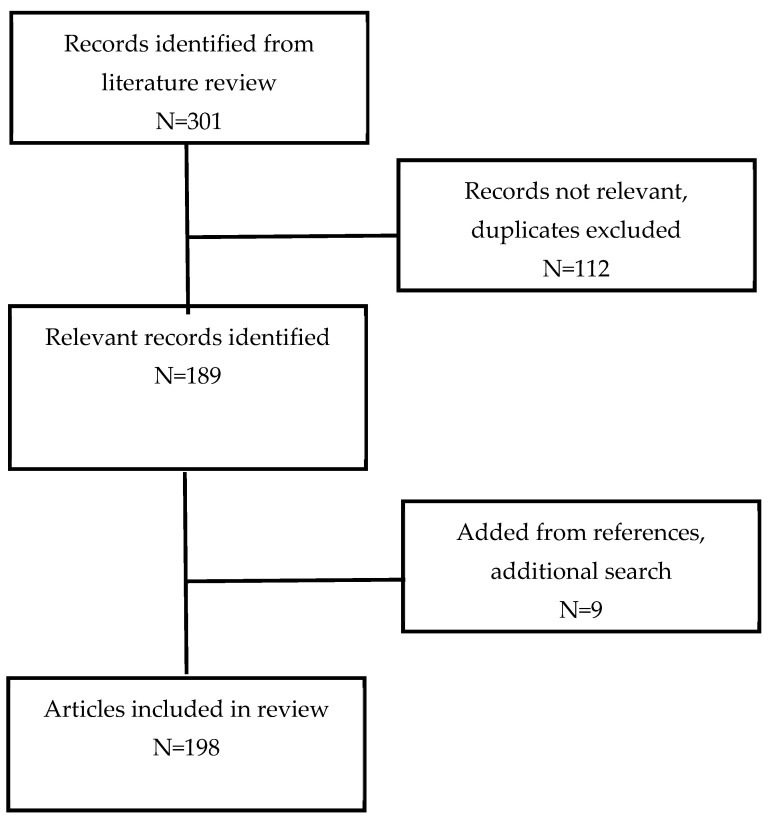
Literature review.

A literature search was carried out by a university librarian at the University of Arkansas for Medical Sciences (UAMS). The search engines used were PUBMED, CINAHL, and Embase. The search terms used were “healthy lifestyle” OR “life style” AND “fertility” OR “preconception care” OR “pre-pregnancy care” OR “pregnancy preparation”. The search was limited to the English language, and the years searched were 1990 to 5/3/2024. The authors independently reviewed the literature search for relevant abstracts. A total of 301 abstracts were identified. The full papers of the abstracts that were relevant to lifestyle modifications or pre-pregnancy preparations in anticipation of becoming pregnant were obtained and read. After removing duplicate papers and papers not relevant to this review, we determined that 189 articles contained applicable information for this review. The references for these full articles were examined for any additional applicable articles. A final total of 198 articles were identified and became the basis of this review (Figure 1).

## 3. Results

### 3.1. Effect of Lifestyle Modifications on Pregnancy Outcomes

Even though PCC and lifestyle modifications are acknowledged as having a beneficial impact, evidence of their effect on live births remains mixed. Some studies suggest that PCC and preconception lifestyle modifications have no influence on the live birth rate or perinatal outcomes [6,7]. Other interventions including the addition of folic acid to the diet and reducing alcohol use have shown significant behavioral improvements when implemented prior to and in early pregnancy [8]. In one comparative study, adverse birth outcomes were significantly less frequent in the group receiving PCC [9]. Despite the acknowledgement of the benefits of PCC, nonpregnant women exhibit markedly lower adherence to lifestyle modifications and guidelines compared to pregnant women regardless of pregnancy risk as determined by contraceptive use status [10]. Surveys across Italy and the Netherlands demonstrate a very high prevalence of risk factors for adverse pregnancy outcomes in non-pregnant women [11,12].

Maternal preconception lifestyle interventions have been shown to profoundly impact their children’s cardiovascular and metabolic health, particularly in obese populations [13]. The offspring of women with obesity who participated in preconception interventions demonstrated improved cardiovascular health (higher left ventricular ejection fractions and less interventricular septal thickening). These findings suggest improved cardiac function and structure [14]. Higher vegetable and fruit consumption in a Dutch study of overweight and obese women correlated with lower diastolic blood pressure and arterial stiffness in their children [15]. Other studies have demonstrated that following a healthy preconception lifestyle, which was defined as maintaining a healthy BMI, smoking cessation, ≥150 min/week of physical activity, consuming a nutritious diet, and folic acid supplementation resulted in a reduction of offspring obesity risk by up to 75% [16], significantly lowered the risk of hypertension and pregnancy, gestational diabetes mellitus (GDM), and small-for-gestational-age (SGA) infants [17]. Additionally, maintenance of low stress levels and a healthy diet during the preconception period/early pregnancy was linked with a 70% reduction in preterm birth (PTB) [18]. In a trial of preconception blood pressures and reproductive outcomes, each 10 mmHg increase in preconception diastolic blood pressure increased the subsequent risk of pregnancy loss by 18% [19].

While some high risk behaviors such as smoking and alcohol may be reduced in pregnancy, dietary habits often remain unchanged, particularly among younger women with less education [20]. Diets abundant in fruit, nuts, and low-fat dairy and particularly when combined with physical activity, have been correlated with reduced GDM risk [21,22]. In one study, higher healthy eating Index scores were related to increased birthweights after adjustment for gestational age and independent of the maternal BMI [23]. Childhood immune function and allergies are affected by the maternal diet. Maternal consumption of low and high-fat dairy products and fresh fruit has been shown to reduce childhood eczema, wheezing, and rhinitis; while diets high in poultry and fruit juice were linked with adverse immune outcomes [24].

The world-wide increase in obesity during pregnancy and its link to metabolic syndrome in the offspring of those pregnancies is very concerning [25,26]. Many of the women who are now becoming pregnant are overweight or obese. Many of these women have sub-optimal behavior in relation to their weight and limited engagement with health care professionals [27] despite high self-reported interest in preconception weight management [28]. Preconception weight loss improves fertility, metabolic function, reduces inflammatory profiles [29,30], and may help reverse intergenerational trends in obesity [31]. An example of reversing this trend is a study who followed 262 children whose mothers participated in gestational weight gain restriction programs; these children had a lower BMI at age five compared to younger siblings [32].

Smoking, alcohol, and substance use prior to, and during, early pregnancy increases adverse maternal and perinatal outcomes. Women with unplanned pregnancies are more likely to smoke and consume alcohol during pregnancy compared to those whose pregnancies were planned [33]. A third of Danish women in one study, despite widely publicized recommendations, reported binge drinking and smoking during early pregnancy [34]. Smoking cessation prior to pregnancy is important because it reduces the risk of fetal growth restriction, GDM, and hypertension [17]. Alcohol cessation prior to pregnancy is important as it is linked to early pregnancy loss, fetal alcohol syndrome, fetal growth restriction and other adverse neurodevelopmental outcomes in children [35]. During pregnancy, a mother’s future fertility and fecundability may be affected by weight, vitamin and iodine intake, alcohol and caffeine consumption, smoking, substance abuse, stress, environmental pollutants, vaccinations, and oxidative stress [36,37]. In IVF settings, it has been observed that smoking and high BMI independently and cumulatively impair the quality of the oocyte [38]. Maternal caffeine intake (>300 mg/day) is associated with a 31% higher risk of fetal loss, preconception alcohol consumption is associated with a 30% increase in the risk of a spontaneous abortion and smoking nearly triples the risk of congenital heart defects [35].

Although it is known that physical activity improves reproductive health, maternal outcomes, and mental well-being before, during, and after pregnancy, fewer than half of women in one study met the recommended activity levels [39]. Higher participation rates in the physical activity recommended for pregnancy improves fertility and sexual function [40,41]. Physical activity also reduces fasting and post-load glucose levels leading to a significantly lowered risk of GDM [22,42,43] and PTB [44]. Postpartum mental health reflected benefits in women who were physically active before and during pregnancy, as 34% were less likely to report anhedonia following pregnancy [45].

Management of mental health disorders are fundamental components of PCC as they affect fertility, pregnancy outcomes, and postpartum well-being [41,46]. Fecundability has been shown to be reduced 46% by stress [47]. Additionally, preconception stressful life events are correlated with increased tobacco and alcohol use before and during pregnancy with subsequent risk of lower neonatal birth weight [48,49]. Mental health disparities are frequently encountered during the preconception period. Foreign born women in Sweden reported lower alcohol and tobacco use compared to Nordic-born women, but they were more likely to report depressive symptoms [50]. Beneficial lifestyle changes during pregnancy are achieved when positive attitudes and personal values are aligned with health priorities [51].

### 3.2. Evidence for Preconception Lifestyle Modifications

#### 3.2.1. Weight Management

The increasing number of women who are overweight/obese and their associated increased risk for maternal and perinatal morbidity and mortality create significant challenges since weight reduction before pregnancy can improve health, fertility, and pregnancy outcomes [52,53,54]. Survey data reveals that women recognize the importance of preconception weight reduction and are interested in dietary counseling, weight reduction, and participating in physical activity [28,55]. Despite this interest and recommendations for lifestyle interventions, particularly those focusing on diet and exercise, evidence regarding their effectiveness remains uncertain [56].

In women with infertility, interventions such as structured dietary and physical activity have improved fertility compared to infertility treatment alone [57,58].

Nonpharmacologic interventions including very low-energy diets [54] and habit-forming mobile applications combined with motivational interviewing [59] have been found to be helpful for preconception obesity management [15]. One meta-analysis demonstrated improved natural conception rates and pregnancy duration, but reported no significant effect on the live birth rate and an observed an increased rate of miscarriage [60]. Similarly, one randomized controlled trial showed significant weight loss with an associated increase in ovulation rates in the lifestyle modification intervention group, but no significant effect on live birth rates [61].

Sedentary behaviors among reproductive-age women present significant physical and mental health risk to those preparing to conceive. In a Canadian survey, only 5% of respondents met all recommended guidelines for physical activity, sedentary behavior, sleep, and screen time. Women who were obese, depressed, and had poor self-perceived health were associated with lower compliance for the guidelines [62]. An Ethiopian study observed that among reproductive-age women, watching television at least once a week increased the odds of being overweight and obese, especially if they resided in urban areas, had a higher education, or belonged to higher wealth categories [63].

Cultural and social influences can also shape physical activity. In Iran, observational learning emerged as the strongest predictor of moderate-to-vigorous physical activity in women preparing for pregnancy [64].

#### 3.2.2. Nutrition Supplementation

Maternal nutrition including diet and micronutrient supplementation plays a critical role in lifestyle modifications prior to and during pregnancy.

Folic acid is essential before and during pregnancy to prevent congenital neural tube defects. Daily supplementation with 400 mcg reduces the incidence of NTDs by up to 72% [65]. Additional protection to prevent open neural tube defects is observed at higher intake levels of dietary and total folate [65]. Despite the information available linking folate insufficiency and open neural tube defects, approximately 3000 babies are born annually with these defects [66,67]. Furthermore, studies have shown that higher pre-pregnancy folate intake reduces the risk of GDM even after adjustments for other micronutrient intake [68]. Folic acid levels have been shown to lower the risk for autism spectrum disorders, while air pollution had been shown to increase the risk for autism spectrum disorders [69].

Folic acid supplementation during preconception and early pregnancy remains an underutilized intervention despite its well-known benefits in preventing neural tube defects (NTDs). Only 30% of women aged 18 to 24 take folic acid supplements, and only 6% understand the appropriate timing for supplementation [67]. Preconception folic acid supplementation remains at a low level across multiple countries: 16.8% in Italy, 33.6% in Lebanon, and 56% in Belgium with similar low levels observed in Spain, Australia, and the U.S. [70,71,72,73,74,75,76]. Even women with a history of open neural tube defect often fail to meet folic acid intake guidelines [77]. Obstacles include unplanned pregnancies, lower educational attainment, and substance use; while a higher level of folic acid use is observed among older women and those with earlier pregnancy detection [67,77,78].

In addition to folic acid supplementation, other recommendations for diet and micronutrients are summarized in Table 1.

#### 3.2.3. Management of Preexisting Diabetes and Gestational Diabetes

Early and effective management of preexisting diabetes (Type 1 [T1DM] or Type 2 [T2DM]) is critical to improving both maternal and fetal outcomes.

These pregnancies are at risk for congenital anomalies, stillbirth, large-for-gestational-age (LGA) infants, and maternal complications of hypertension and nephropathy [87,88,89,90,91]. Tight glycemic control through diet, physical activity, and insulin therapy, when necessary, is essential for optimal maternal/fetal outcomes. In pregnancies complicated by T1DM, intensive insulin regimens via multiple daily injections, continuous subcutaneous insulin infusion, or hybrid closed-loop systems assist in reducing hypoglycemia and maintain stability of insulin requirements as they fluctuate throughout pregnancy [92]. Insulin analogs like insulin lispro and insulin aspart offer further control and are pregnancy compatible [93].

Adolescent T1DM pregnancies frequently have poor glycemic control, often underestimating the consequences of uncontrolled diabetes on pregnancy and fetal development. This is emphasized in a study where 20% of respondents reported being sexually active, nearly 25% had HbA1c levels above 9%, and half fell between 7.5–9% [94]. In contrast, women with T2DM may underestimate the severity of their diabetes when pregnant, but often have the added challenges of obesity, PCOS, sedentary lifestyles, and barriers to early pregnancy healthcare. Metformin therapy can improve ovulatory function in PCOS, while transitioning to insulin prior to conception further supports glycemic optimization [95].

Preconception glycemic control in T1DM or T2DM is highlighted in a population-based study from Ontario. For each 0.5% net decline in HbA1c, the congenital anomalies rate was lowered by 6% overall, reduced cardiac anomalies by 11%, reduced PTB risk by 11%, and decreased severe maternal morbidity or death by 10% [96]. GDM affects 6–7% of pregnancies in the US and UK and increases the likelihood of LGA infants, posing significant risks for both mother and child [90,97]. Women with a history of GDM face a recurrence risk of 40–73% in a subsequent pregnancy with a significantly increased lifetime risk of developing T2DM, metabolic syndrome and cardiovascular disease. This risk is not just limited to the mother, but also extends to their offspring [97,98].

Management of these risks includes early screening for GDM in at-risk pregnancies (women with impaired fasting glucose, normoglycemic obesity, or a prior history of GDM [99]. Postpartum glucose screening is currently strongly encouraged for all women diagnosed with GDM [100,101,102]. GDM can now be detected sooner in at-risk pregnancies allowing for earlier classification. One identified model integrated four features: HbA1c, mean arterial blood pressure, fasting insulin, and triglycerides-to-HDL ratio labeling these as strong predictors for GDM and an increased risk for preterm delivery [103]. Likewise another model featured five clinical risk factors—race/ethnicity, age at delivery, pre-pregnancy BMI, family history of diabetes, preexisting hypertension—and classified women as at risk for GDM; this model has been validated [104].

When lifestyle interventions fail to maintain glycemic control, the gold standard treatment is insulin. If the patient is unable or unwilling to take insulin, metformin and glyburide are viable oral substitutes [105]. In the RADIEL trial conducted in Finland, women with a history of GDM and/or a pre-pregnancy BMI ≥ 30 kg/m^2^ (intervention group) had a 39% reduction in their risk of developing GDM pursuing diet, physical activity, and weight-management counseling in addition to standard prenatal care [106]. The National Diabetes Prevention Program has reported decreased periconceptional obesity and hyperglycemia early in pregnancy among a diverse, low-income group of women in the United States [52]. Other health strategies emphasize postpartum and between-pregnancies screening for diabetes, regular cardiometabolic monitoring, long-term contraception for optimal pregnancy timing, and breastfeeding support [98,102]. The CDC and a number of other organizations have endorsed similar measures to lessen the long-term disease burden of diabetes [52,107].

The goal is to incorporate preconception counseling, early diabetic screening, lifestyle changes, and ongoing management during postpartum and between pregnancies to effectively address women with T1DM, T2DM, or GDM and improve maternal and fetal health [108,109,110].

#### 3.2.4. Smoking, Alcohol, Substance Use and Environmental Exposures

Exposure to caffeine, alcohol, and substance use -both during preconception and pregnancy—increase the risks for pregnancy fetal growth restriction, congenital anomalies and developmental disorders [35]. Community and policy level interventions targeting these modifiable risk factors have proven effective. One example of the benefits of targeting these behaviors was a study of over 41,000 Chinese women. This study emphasized avoiding secondhand smoke, reducing alcohol consumption, and managing stress; improvements were observed in the patient’s diet and in their level of physical activity [111]. In a study evaluating maternal smoking patterns, education, poverty status, and depression investigators categorized participants as persistent smokers, temporary quitters, and delayed initiators, effectively identifying women at high risk and more in need of directed intervention [112]. Government led initiatives have also been used.

A Dutch campaign increased the odds of reducing alcohol use prior to pregnancy by 72% [113], and a Shanghai initiative offered free pre-pregnancy health assessments resulting in improvements in folic acid supplementation, smoking cessation, and alcohol abstinence [114].

Integrating lifestyle modifications into preconception counseling during routine clinical care is beneficial. A personalized program of preconception interventions resulted in 30% of smokers to quit smoking and 50% of patients with obesity to lose an average of 6.1 kg before IVF treatment [115]. In women with inflammatory bowel disease, PCC improved medication adherence, folic acid intake, smoking cessation, and significantly lowered the risk of IBD relapse and low birth weight in their offspring [116].

Digital tools and mobile health platforms assist in dealing with access issues and barriers in PCC by changing the behaviors of smoking and alcohol consumption in pregnancy [117,118]. These studies’ “Smarter Pregnancy” program improved dietary scores and increased pregnancy rates, particularly in underserved communities and when male partners were engaged [119,120,121,122]. The success of these programs have inspired programs elsewhere, such as the “Jom Mama” program planned in Malaysia [123].

Web-based questionnaires like Zwangerwijzer.nl and reproduktivlivsplan.se have been well received by both users and clinicians for enabling preconception pregnancy risk assessments and fertility planning [124,125]. Adapted digital education, as demonstrated in a cohort of Italian women, improves folic acid use and decreases alcohol intake [126], though gaps remain in information obtained between public search behavior and evidence-based information [127].

Environmental exposures occurring in pre-conception and in early pregnancy affect fetal and neurodevelopmental health. Environmental contaminants (air particulates, heavy metals, and endocrine-disrupting chemicals (EDCs) are connected to an increased risk of autism spectrum disorder (ASD) and adverse perinatal outcomes [3,69]. While many maternity care providers lack formal training in environmental toxicology, this ‘window of opportunity’ can be leveraged for anticipatory guidance [128]. For example, findings from the TIDES cohort suggest that phthalate exposure, a known endocrine disrupting chemical, does not significantly differ between planned and unplanned pregnancies [129]. Other areas that could be environmental risks include living in high HIV prevalence areas or in malaria-endemic areas. Introducing early interventions, such as antiplatelet agents for pre-eclampsia prevention may reduce the risk of SGA infants for these at-risk populations [130].

#### 3.2.5. Maternal Mental Health and Stress Management

Mental health and stress assessments in the preconception period or in early pregnancy can improve both maternal and child health outcomes. In a systematic review, seven key factors (knowledge, beliefs about capabilities and consequences, goals, intentions, social support, and environmental context) were recognized as influencing women’s external preconception behaviors of diet, physical activity, smoking, alcohol use, and supplement intake [131]. In these women, cognitive-behavioral therapy (CBT) could be particularly effective. In women with polycystic ovary syndrome, management consisting of weight loss and oral contraceptive therapy has been shown to improve health-related life conditions and decrease the symptoms of depression and anxiety [132]. A multidisciplinary CBT intervention over a 12 month period of time combined diet and physical activity, resulting in maintainable 5–10% weight loss in women with PCOS, improved metabolic and reproductive outcomes (including rates of ovulation), and psychological well-being [133,134].

Health education workshops also appear to be helpful. An Iranian randomized controlled trial established the benefits of a short-term workshop. Participants significantly improved their control and self-efficacy related to physical activity [135]. Another study included six group counseling sessions over three weeks and demonstrated significant improvements in preconception lifestyle practices. Women in the intervention group scored higher on lifestyle indices compared to the control group at 4 and 8 weeks post-intervention assessment [136].

#### 3.2.6. Knowledge Gaps in Understanding Lifestyle Factors

Although the benefits of preconception and early pregnancy lifestyle are known, significant knowledge gaps exist in understanding those behaviors and their impact on maternal and perinatal outcomes. Investigations reliably reveal that most couples have lifestyle-related risk factors that require modification prior to a pregnancy, yet many are unaware of how sedentary activity, alcohol use, and inadequate nutritional supplement intake impact pregnancy outcomes [137,138,139]. Australian, Italian, and UK surveys emphasize the inadequate understanding of preconception health, particularly among those who are not actively pursuing fertility treatments [140,141]. Even among women attending infertility clinics, critical knowledge is lacking regarding alcohol consumption, risks related to advanced maternal age, and exposure to infections [142]. Despite these knowledge gaps, preconception consultations have been successful in increasing the use of folic acid during preconception and in reducing binge drinking [143].

#### 3.2.7. Association of Health Beliefs and Pregnancy Planning

Research exposes a disconnect between pregnancy planning and observance of a healthy lifestyle with women feeling ‘healthy enough’, avoiding preconception counseling, and engaging in high-risk behaviors [144,145]. For example, women with mental illness or T2DM are less likely to plan for pregnancy [146]. This lack of pre-pregnancy preparation extends to environmental exposures and lifestyle behaviors [129]. Women with lower pregnancy preparation scores were more likely to engage in binge drinking [34], and the preconception stressors of bereavement and infertility, were associated with higher use of alcohol and tobacco during pregnancy [49]. Women with infertility who track their basal body temperature, evaluate their cervical mucus, and monitor luteinizing hormone levels are much more likely to become pregnant [147]. Although some women have reduced their caffeine use during conception attempts [148], others fall short in engaging in physical exercise and nutritional supplements [149,150,151]. Lifestyle modifications are also less likely in women with socioeconomic disparities and multiparity [152]. In contrast, women actively seeking preconception counseling are more likely to adopt healthier diets, eliminate alcohol consumption, and consume folic acid [153,154].

#### 3.2.8. Education and Peer Support

Educational and peer support may be helpful in increasing participation in lifestyle modifications. Common obstacles that prevent PCC include stress, confusion, and misinformation [155]. When women receive targeted preconception information, they are more likely to stop consuming alcohol and adopt healthier diets [153,156]. It has been observed in younger women that peer-led and group-based programs on college campuses or integrated into existing healthcare visits enhance self-efficacy and preconception engagement [135,157,158,159].

#### 3.2.9. Psychosocial Planning

Psychological planning, especially for the first pregnancy, begins before conception involving maternal mental images of the pregnancy and emotional attachment to the pregnancy as the pregnancy is planned [160]. If prior pregnancies have involved recurrent pregnancy losses, the counseling offers reassurance, support, recommendations for genetic screening, and information about healthy lifestyle modifications [161]. Personal values and the importance of family help shape preconception priorities, with healthier behaviors more likely when parenthood is perceived as an important life goal [51].

### 3.3. Barriers to Pregnancy Planning and Lifestyle Modification

#### 3.3.1. Socioeconomic and Demographic Discrepancies

Socioeconomic status, marital status, and educational levels shape preconception and maternal health behaviors, impacting both lifestyle modification and healthcare access. However, systemic barriers, particularly in high-minority or low-income areas, limit access to educational materials, specialist referrals, and financial resources [162]. In Malawi for instance, proactive pregnancy preparation such as adopting healthier diets and saving money was reported by only 36.1% of women, with married women and those spacing births at least two years apart showing significantly higher preparedness [163]. In countries such as Spain and Belgium, making significant preconception lifestyle changes was found to be less likely among women of younger age, lower social class, those with lower educational attainment, or those facing financial difficulties [73,152]. In West Virginia only 47% of women reported dental cleanings before pregnancy. Although younger, those with private health insurance and women with higher education levels were more likely to receive dental care [164].

#### 3.3.2. Cultural and Ethnic Influences

Low- and middle-income countries (LMICs) face the burden of both inadequate nutrition and obesity. Although there is good evidence that antenatal multiple micronutrient supplementation reduces the risks of stillbirths, low birth weight, SGA births, and childhood malnutrition the treatment prior to and during pregnancy of micronutrient supplementation remains limited in LMICs [165,166] Migrant women in Australia often have limited awareness of preconception and early pregnancy lifestyle modifications, with many pregnancies being unplanned and with language and cultural barriers, this limitation further impedes reproductive health discussions [167]. In the Nordic region, non-European women demonstrate lower alcohol and tobacco use than Nordic-born women but report higher rates of depressive symptoms. Religious beliefs generally correlate with healthier behaviors; although integration levels do not extensively influence lifestyle choices, it underscores the importance of culturally explicit mental health strategies [50]. In another study of foreign-born Latinas, they exhibited healthier dietary examples compared to U.S.-born Latinas and non-Latina whites by consuming fewer calories from fat and with higher intakes of carbohydrates, fiber, folate, and vitamin C in their diet [168]. A separate study noted similar patterns of illicit drug use, binge drinking, smoking and BMI [169]. A distinction is drawn in indigenous groups from Australia, Canada, New Zealand, and the U.S. who experience higher infant mortality rates associated with smoking, alcohol consumption, and inadequate nutrition [170]. Regional differences in the same country are also observed in a study from China where there were significant differences between provinces, with women in Jiangsu reporting better health and higher education levels than those in Hebei [171]. In the U.S. Mississippi Delta, women’s preconception health was poorer than in other Mississippi regions, and were 1.3–1.7 times less likely to meet optimal physical activity and nutrition recommendations [172]. Race and ethnicity along with genetic predisposition, environmental influences and pharmacogenomics affect preterm birth rates [173]. Preventive strategies require a deeper understanding of sociodemographic factors, nutrition, lifestyle habits, and individual genetic variations, with data-driven approaches offering promising directions for reducing PTB risks [173].

#### 3.3.3. Special Populations: Disabilities and Adolescents

Certain populations, including adolescents and women with disabilities face unique barriers to effective PCC. Women with disabilities report higher rates of low education, lower income, insufficient dental care, obesity, current smoking, binge drinking, and physical inactivity [174]. Adolescents, particularly those with conditions like T1DM, are often inadequately informed with little guidance on reproductive health and often have inadequate education on pregnancy-related risks [94,175,176].

#### 3.3.4. Shortcomings in Healthcare Systems, Counseling, and Knowledge

Despite the proven benefits of preconception counseling, many healthcare systems lack a focused approach. Studies have shown significant gaps in women’s awareness of preconception risks, with many relying on informal online sources rather than professional healthcare advice [140,156,177]. Inconsistencies in PCC guidelines between professional organizations and from different countries further complicate access to consistent, reliable information, with specific gaps noted for women of advanced maternal age and other vulnerable groups [127,178,179]. Evidence from surveys of healthcare providers demonstrates focused counseling on lifestyle changes, like diet and exercise, shows promise if implemented [180,181].

#### 3.3.5. Tools for Assessment and Collaborative Approaches

A number of components are essential for a useful PCC including accurate tools, inclusive program design, multidisciplinary care teams, and cross-cultural communication [182,183]. The Preconception Health Knowledge Questionnaire (PHKQ) is a valuable tool due to its high validity in assessing reproductive health knowledge, highlighting the need for education tailored to demographic specifics [184]. Co-design proposals with diverse community input help ensure PCC programs meet the needs of different populations, highlighting the importance of a strong community network and accessible healthcare services [185,186,187].

#### 3.3.6. Summary of Studies on Lifestyle Modification

All studies used in this review have been divided by type of study, intervention/exposure, and findings into four supplemental tables. Supplementary Table S1 is labeled as meta-analysis, Supplementary Table S2 as experimental studies, Supplementary Table S3 as observational studies on specific lifestyle modifications and Supplementary Table S4 as observational studies focused on the importance of lifestyle modifications [Supplementary Tables S1–S4].

#### 3.3.7. Guideline Reviews of Lifestyle Modifications by Professional and National Organizations

Considering the multitude of publications on lifestyle modifications prior to pregnancy, including recommendations (Over 750 in PubMed), a review of guidelines established by national organizations is useful. We searched the websites of the American College of Obstetricians and Gynecologists (ACOG), American Society of Reproductive Medicine (ASRM), French College of Gynecologists and Obstetricians (CNGOF), Fetal Medicine Foundation (FMF), International Federation of Obstetrics and Gynecology (FIGO), Japanese Association of Obstetricians and Gynecologists (JAOG), National Institute for Health and Care Excellence (NICE), Royal Australian New Zealand College of Obstetricians and Gynecologists (RANZCOG), Royal College of Obstetricians and Gynaecologists (RCOG), Society for Maternal-Fetal Medicine (SMFM), and the Society of Obstetricians and Gynaecologists of Canada (SOGC). We conducted a review of guidelines on preconception lifestyle recommendations. Among the organizations examined, three (CNGOF, FMF, and SMFM) did not provide any recommendations. However, nine organizations provided clear guidance on various aspects of preconception health. A summary of these recommendations is outlined in Table 2 [188,189,190,191,192,193,194,195].

## 4. Discussion

**Figure 2:**
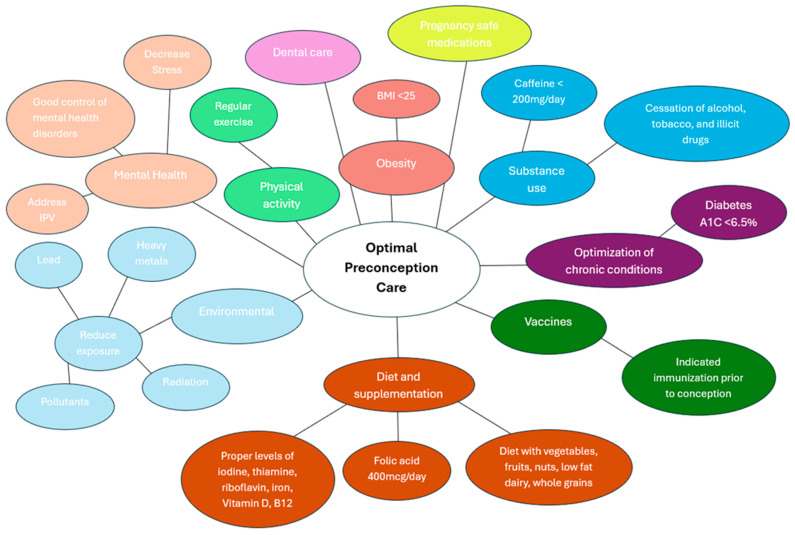
Key Domains of Preconception Care.

Achieving optimal preconception health requires a holistic, integrative approach that supports the physical, emotional, nutritional, environmental, and social well-being of both prospective parents. Rather than viewing individual health behaviors in isolation, this framework emphasizes lifestyle, mental health, partner involvement, and structural access to care. A summary diagram (see Figure 2) visually consolidates these preconception recommendations across physical, nutritional, behavioral, environmental, and psychosocial domains.

Optimal metabolic health begins with achieving and maintaining a healthy body mass index of 18.5–24.9 prior to conception. For overweight or obese individuals, structured weight-loss programs that combine diet and physical activity under supervision are recommended. Women with high BMI or a history of GDM should consider enrollment in structured interventions such as lifestyle coaching or the National Diabetes Prevention Program (NDPP). This focus is informed by evidence linking high pre-pregnancy BMI to increased risks of GDM, hypertensive disorders, macrosomia, cesarean sections, and long-term obesity in offspring. Such interventions not only improve metabolic markers but also enhance ovulation and pregnancy rates; although, the impact on live birth outcomes may vary.

Nutritional recommendations emphasize early initiation of folic acid supplementation (400–800 mcg/day) at least one month before conception to significantly reduce the risk of neural tube defects. Prospective mothers are advised to adopt a nutrient dense, anti-inflammatory diet such as the Mediterranean or DASH diets that prioritize fruits, vegetables, whole grains, lean proteins, and healthy fats while avoiding sugary beverages and processed foods. Screening for and addressing deficiencies in key micronutrients like iodine, vitamin D, iron, and B12 are also critical. This nutritional strategy is backed by evidence showing that a quality preconception diet can mitigate the risk of GDM, influence fetal growth, and improve cardiovascular outcomes in offspring, and have been shown to enhance the success of assisted reproductive technology.

Physical activity, an integral component of preconception care, is recommended at a threshold of at least 150 min of moderate-intensity activity per week. Activities should promote cardiovascular fitness, strength, and flexibility. Efforts should be made to reduce sedentary behaviors, particularly excessive screen time, and encourage movement throughout the day. The benefits of such activity extend beyond physical health, improving mental health and fertility, and reducing the risks of GDM and depressive symptoms during pregnancy.

Recommendations strongly advocate for abstaining from tobacco, alcohol, and recreational drugs, with early screening for substance use and support for cessation. Additionally, avoiding exposure at home or in occupational environments to endocrine-disrupting chemicals, heavy metals, and secondhand smoke is imperative. Smoking and alcohol use not only elevate the risk of miscarriage and SGA infants but also contribute to GDM and adverse neurodevelopmental outcomes in children, while environmental toxins have been linked to increased rates of infertility and autism spectrum disorders. While preconception cessation of vape use is recommended, further research needs to be done on its preconception use and effects on pregnancy outcomes.

Mental health screenings for depression, anxiety, and high stress levels are essential during preconception visits with options for counseling, peer support, or cognitive-behavioral interventions provided as needed. Stress management practices such as mindfulness, regular exercise, and maintaining good sleep hygiene are recommended to support mental well-being. Research demonstrates that psychological distress can diminish fecundability and elevate risky pregnancy behaviors, whereas mental health interventions can substantially improve both behavioral adherence and postpartum outcomes.

Effective preconception care includes a comprehensive review of medications, with an aim to discontinue any teratogenic drugs while continuing effective management of chronic conditions such as diabetes, thyroid disease, and hypertension. Employing a reproductive life plan can guide the timing and intentions surrounding pregnancy by enhancing preparedness and improving health behaviors.

Involving male partners in preconception health efforts is vital, including counseling for smoking cessation, alcohol moderation, and weight management. Addressing paternal health optimizes fertility and improves offspring health outcomes. Evidence indicates that male BMI, smoking, and alcohol use can negatively impact sperm quality, time to pregnancy, and child health, while couple-based interventions are known to improve adherence to health recommendations and overall outcomes [6].

Integrating preconception counseling into routine visits with primary care providers, OB/GYNs, endocrinology, behavioral health specialties, and family planning clinics is essential. Leveraging digital tools and community outreach can enhance awareness, particularly among underserved groups, adolescents and other at-risk groups. Interventions should be tailored to meet diverse cultural, socioeconomic, and language needs.

While this synthesis offers insight into preconception health strategies, several limitations should be considered. Heterogeneity among included studies, such as differences in population characteristics, intervention delivery, outcome definitions, and follow-up durations, can reduce generalizability of findings. Publication bias may skew results toward more favorable outcomes, particularly if null or negative studies remain unpublished. Furthermore, many included studies rely on observation data which may limit the ability to infer causality and the ability to draw definitive conclusions. Critically, there is a notable gap in high-quality, large-scale randomized controlled trials specifically targeting the preconception period, especially those evaluating long-term offspring outcomes and real-world implementation strategies. Addressing these gaps in research is essential to develop equitable, evidence-based models of care that support all individuals preparing for pregnancy.

Optimizing health prior to conception through targeted interventions spanning nutrition, physical activity, mental wellness, substance avoidance, optimization of chronic conditions, environmental safety, and chronic disease management plays a pivotal role in enhancing fertility, pregnancy outcomes, and long-term health outcomes for both parents and offspring. These strategies are most effective when approached holistically. A comprehensive and inclusive preconception care model that integrates clinical guidance with culturally sensitive, community-based, and partner-inclusive approaches has the potential to meaningfully reduce adverse reproductive outcomes and promote intergenerational health.

## Figures and Tables

**Table 1:** Diet, Supplementation, and Micronutrient Recommendations.

Nutrient	Recommendation	Affected Outcome	Compliance	Additional Comments
A nutrient rich diet includes whole grains, fruits, vegetables	No specific recommendation	Associated with improved fetal health, normal birth weight, and improved maternal and infant survival rates [79,80,81,82]	No data	Diets high in sugar and fat predispose women to diabetes, metabolic syndrome, and cardiovascular disease [79,80,81,82]
Iodine	≥150 µg/day at least four weeks prior to conception	Significantly lower serum thyroglobulin levels at 16 weeks [83]	No data	
Calcium	No specific recommendation	Vital for fetal development	No data	
Vitamin C	Particularly recommended for patients who continue to smoke [81]	Lowers incidence of asthma and wheezing in offspring [81]	No data	
Vitamin B12	No specific recommendation	In combination with “pro-fertility diet” outperformed other popular diets in enhancing live birth [84]	No data	Described as part of “pro-fertility diet” [83]
Vitamin D	No specific recommendation	In combination with “pro-fertility diet” outperformed other popular diets in enhancing live birth [84]	No date	Described as part of “pro-fertility diet” [83]
“Pro-fertility diet”	Emphasizes folic acid, vitamin B12, vitamin D, low-pesticide fruits and vegetables, whole grains, dairy, soy, and seafood (over other meats) [84]	This regimen has outperformed other popular diets in enhancing live birth rates in women undergoing assisted reproductive technology therapy [84]	Despite this observation, real-world adherence remains limited [85]	In one sub-fertile outpatient cohort, most couples initially failed to meet their dietary targets. After counseling, fruit and fish intake was increased, alcohol use declined, and overall dietary risk scores improved in both women and men [86]

**Table 2:** Preconception lifestyle recommendations by professional organizations [188,189,190,191,192,193,194,195].

Resource	Country	Preconception Recommendations
ACOG	United States	Follow a healthy diet (MyPlate as a guide); take a prenatal vitamin with 400 mcg folic acid and iron Exercise regularly (150 min/week; include muscle-strengthening exercises 2+ days/week) Achieve and maintain a healthy BMI Avoid unhealthy substances (tobacco, alcohol, marijuana, illegal drugs, nonmedical prescription drugs) for both partners Ensure a safe environment (check for lead, mercury, pesticides, and radiation) Manage chronic health conditions Review medications during PCC visits Get recommended vaccinations before pregnancy Plan pregnancies with appropriate intervals
ASRM	United States	Optimize management of chronic conditions (e.g., hypertension, diabetes, obesity, thyroid disorders, psychiatric disorders, HIV, thrombophilias, autoimmune diseases, cardiopulmonary disorders) Review medications for safety, efficacy, and potential dosing adjustments Collect detailed family history, provide genetic counseling, and offer screening Assess immunization status and provide necessary vaccinations Ensure cervical cancer screening is current Evaluate the need for STI screening and treat any infections found Screen for substance use and exposure to intimate partner violence (IPV) Screen for teratogenic environmental or occupational exposures Discuss safe interpregnancy intervals for patients with recent deliveries Review past pregnancy complications, assess recurrence risks, and discuss mitigation strategies
CNGOF	France	No recommendations
FIGO	United Kingdom	Manage pre-existing medical conditions Maintain a healthy weight (address obesity/low weight) Take folic acid and micronutrient supplements Quit smoking Avoid alcohol and substance use Limit exposure to environmental chemicals Engage in regular physical activity (150 min/week) Check immunization status Ensure adequate interpregnancy intervals (12–24 months)
FMF	United Kingdom	Refers to FIGO’s recommendations.
JAOG	Japan	Recommend preconception folate supplementation of 400 micrograms/day
NICE	United Kingdom	Plan your reproductive life (number and timing of pregnancies based on values and goals) Adopt a healthy diet: increase fiber by 10 g, reduce sugar-sweetened beverages, limit red/processed meats and animal fats, and replace animal protein with plant-based options Increase folic acid intake (0.4 mg daily) Update vaccinations (consult your healthcare provider) Discuss any medical conditions (diabetes, hypertension, etc.) with your provider Avoid smoking, alcohol, drugs; seek support if needed Achieve a healthy weight before pregnancy Learn your family health history Prioritize mental health to minimize risks during and after pregnancy
NHS	United Kingdom	Exercise regularly; aim for 20 min of walking after meals and engage in light activities like swimming, antenatal yoga, or low-impact exercises Avoid alcohol Start pelvic floor exercises to support your baby and prevent incontinence post-birth Eliminate smoking, vaping, and exposure to second-hand smoke Consult your GP about any medications you are taking to ensure they are safe during pregnancy Take a daily 400 mcg folic acid supplement and ensure proper intake of iron, vitamin D, iodine, and other essential nutrients Focus on healthy foods ich in folic acid, iron, and iodine, limit high-fat and high-sugar foods, and stay hydrated Practice good hygiene by washing hands, cleaning surfaces, and being cautious around animals Maintain oral health by brushing twice daily with fluoride toothpaste and visiting the dentist regularly Wear seatbelts, plan travel carefully, ensure you have necessary vaccinations, and consider the quality of medical care in your destination
RANZCOG	Australia	Evaluate medical problems and their impact on pregnancy; stabilize existing conditions and assess mental health Offer genetic counseling and screening for heritable disorders based on history or ethnic background Check and maintain vaccinations for SARS-CoV-2, measles, mumps, rubella, varicella zoster, diphtheria, tetanus, and pertussis. Consider immunizations for those with incomplete immunity (Hepatitis B, rubella, varicella) Encourage steps to achieve a healthy BMI through diet, exercise, or bariatric surgery if appropriate Recommend taking 0.4 mg of folic acid daily for at least one month before conception and for the first three months of pregnancy Advise cessation of smoking, alcohol, and substance use before conception; inform there is no safe level of alcohol during pregnancy Advise couples to avoid travel to areas with infections during conception attempts; assess and minimize exposure to toxins or radiation at home and work
RCOG	United Kingdom	Base meals on starchy foods (potatoes, bread, rice, pasta), preferably wholegrain Consume at least five portions of different fruits and vegetables daily Minimize consumption of fried foods Limit drinks with added sugars and foods like sweets, cakes, and biscuits; opt for fiber-rich foods (oats, beans, lentils, grains, seeds) Include protein daily; choose lean meats and aim for two portions of fish weekly. Alternatives include lentils, beans, nuts, and eggs Opt for healthier snacks like vegetables, small sandwiches, or fresh/dried fruit Include dairy or calcium-fortified unsweetened dairy alternatives Be mindful of portion sizes; avoid “eating for two” Aim to eat breakfast daily Limit caffeine to less than 200 mg per day Take an additional folic acid supplement of 400 micrograms daily to reduce the risk of birth defects Take 10 micrograms of vitamin D during pregnancy and breast feeding
SMFM	United States	No recommendations
SOGC	Canada	Inactive vaccines (e.g., influenza, hepatitis B, tetanus, diphtheria, pertussis) are safe in pregnancy. Live-attenuated vaccines (e.g., MMR, varicella) are not recommended due to potential risks. Consider vaccinations before pregnancy Adopt a healthy diet prior to conception to manage obesity and promote a healthy pregnancy Discuss mental health with your healthcare provider to create a plan for reducing risks during and after pregnancy Engage in at least 150 min of moderate-intensity physical activity weekly, aiming for activity 3 to 7 days a week. Include aerobic exercises and resistance training (e.g., walking, swimming, low-impact aerobics, cycling, strength training)

ACOG = American College of Obstetricians and Gynecologists; ASRM = American Society for Reproductive Medicine; CNGOF = The French College of Obstetricians and Gynecologists; FIGO = International Federation of Gynecology and Obstetrics; FMF = Fetal Medicine Foundation; JAOG = Japan Association of Obstetricians and Gynecologists; NICE = National Institute for Health and Care Excellence; RANZCOG = Royal Australian and New Zealand College of Obstetricians and Gynaecologists; RCOG = Royal College of Obstetricians and Gynaecologists; SMFM = Society of Maternal Fetal Medicine; SOGC = Society of Obstetricians and Gynaecologists.

## Supplementary Materials

The following supporting information can be downloaded at: https://www.mdpi.com/article/10.3390/jcm14186582/s1, Supplementary Table S1: Meta-Analysis; Supplementary Table S2: Experimental Studies; Supplementary Table S3: Observational Studies on Specific Lifestyle Modifications; Supplementary Table S4: observational Studies Focused on the Importance of Lifestyle Modifications.
