# Supplementary material for: Lifestyle Modifications Prior to Pregnancy and Their Impact on Maternal and Perinatal Outcomes: A Review
Source: J Clin Med. 2025 Sep 18;14(18):6582. doi: 10.3390/jcm14186582 (PMC12470430; doi:10.3390/jcm14186582)
Supplement: Supplementary file 1 [file jcm-14-06582-s001.zip › jcm-3822662-supplementary.pdf]

Supplement Table 1. Meta-analyses.

Study	PMID	Title	Number of included studies	Interventions/Exposures	Findings
Yu et al. 2022	34459054	Association between pregnancy intention and smoking or alcohol consumption in the preconception and pregnancy periods: A systematic review and meta-analysis.	23	Planned vs. unplanned pregnancy & smoking, alcohol use (preconception & during pregnancy)	Women with unplanned pregnancy had 68% higher odds of cigarette use (OR = 1.68, 95% CI = 1.44-1.95) & 44% higher odds of alcohol use alcohol (OR = 1.44, 95% CI = 1.15-1.81) during pregnancy; 30% higher for smoking (OR = 1.30, 95% CI = 1.10-1.53) & 20% higher for alcohol (OR = 1.20, 95% CI = 1.01-1.42) preconception.
Mijatovic-Vukas et al. 2018	29849003	Associations of Diet and Physical Activity with Risk for Gestational Diabetes Mellitus: A Systematic Review and Meta-Analysis.	40	Diet (Mediterranean, DASH, AHEI patterns; specific foods) & physical activity before/early pregnancy	MedDiet/DASH/AHEI lowered relative GDM risk by 15–38%.<br>Higher pre/early pregnancy physical activity led to 21–30% reduced GDM odds, (OR = 0.70, 95% CI = 0.57–0.85; OR = 0.79, 95% CI = 0.64–0.97) & >90 min/week leisure physical activity pre-pregnancy led to 46% reduced odds of GDM (OR = 0.54, 95% CI = 0.34-0.87).<br>Frequent potato, meat/processed meats, or high animal-protein intake increased GDM risk.
Kim et al. 2020	33066039	Effectiveness of Non-Pharmacological Interventions for Overweight or Obese Infertile Women: A Systematic Review and Meta-Analysis.	21	Nonpharmacological (diet, exercise, counseling, etc.) in overweight/obese infertile women	Increased overall pregnancy rate (RR=1.37; 95% CI 1.04–1.81) & natural conception rate (RR=2.17; 95% CI 1.41–3.34).<br>No significant effect on live birth rate (RR, 1.36, 95% CI, 0.94-1.95; p=0.10), but elevated miscarriage risk (RR: 1.57, 95% CI, 1.05-2.36).
Lassi et al. 2014	25415846	PCC: caffeine, smoking, alcohol, drugs and other environmental chemical/radiation exposure.	39	Preconception caffeine, tobacco, alcohol, illicit drug use, and certain environmental chemicals	Heavy caffeine intake was associated with increased fetal loss risk, >300mg/day<br>(RR 1.31; 95% CI: 1.08-1.58) >420mg/day (RR 6.11;<br>95% CI: 5.12-7.29)<br>>900mg/day (RR 1.72; 95% CI:<br>1.00-2.96).<br>Preconception alcohol associated with nonsignificant spontaneous abortion risk (RR 1.30; 95% CI: 0.85-1.97).<br>Preconception counselling associated with significant decrease in first trimester alcohol consumption (OR 1.79; 95% CI: 1.08-2.97).<br>Periconception smoking associated with almost 3x increased risk of congenital heart defects (OR 2.80; 95%

					CI 1.76-4.47).<br>Limited data on environmental exposures; some occupational radiation exposure linked to miscarriage.
Boedt et al. 2021	33914901	Preconception lifestyle advice for people with infertility.	7	Preconception lifestyle advice (on various topics, e.g., weight, alcohol, smoking, etc.) vs. routine care in infertile patients	Preconception lifestyle advice on a combination of topics affects lifestyle behavioral changes: body mass index (BMI) (mean difference (MD) -1.06 kg/m(2), 95% CI -2.33 to 0.21; 1 RCT, 180 participants), vegetable intake (MD 12.50 grams/d, 95% CI -8.43 to 33.43; 1 RCT, 264 participants), alcohol abstinence in men (RR 1.08, 95% CI 0.74 to 1.58; 1 RCT, 210 participants), or smoking cessation in men (RR 1.01, 95% CI 0.91 to 1.12; 1 RCT, 212 participants).<br>"Preconception lifestyle advice on a combination of topics may result in little to no difference in the number of women with adequate folic acid supplement use (RR 0.98, 95% CI 0.95 to 1.01; 2 RCTs, 850 participants; I(2) = 4%), alcohol abstinence (RR 1.07, 95% CI 0.99 to 1.17; 1 RCT, 607 participants), and smoking cessation (RR 1.01, 95% CI 0.98 to 1.04; 1 RCT, 606 participants)"<br>"Compared to routine care, we are uncertain whether preconception lifestyle advice on weight affects the number of live births (RR 0.94, 95% CI 0.62 to 1.43; 2 RCTs, 707 participants; I(2) = 68%; very low-quality evidence), adverse events including (RR 0.78, 95% CI 0.48 to 1.26; 1 RCT, 317 participants; very low-quality evidence), hypertension (RR 1.07, 95% CI 0.66 to 1.75; 1 RCT, 317 participants; very low-quality evidence), or miscarriage (RR 1.50, 95% CI 0.95 to 2.37; 1 RCT, 577 participants; very low-quality evidence<br>Insufficient data on adverse events, miscarriage."<br>"Regarding lifestyle behavioral changes for women with infertility and obesity, preconception lifestyle advice on weight may slightly reduce BMI (MD -1.30 kg/m(2), 95% CI -1.58 to -1.02; 1 RCT, 574 participants; low-quality evidence)."<br>"We are uncertain whether preconception lifestyle advice on alcohol intake affects the number of live births (RR 1.15, 95% CI 0.53 to 2.50; 1 RCT, 37 participants; very low-quality evidence) or miscarriages (RR 1.31, 95% CI 0.21 to 8.34; 1 RCT, 37 participants; very low-quality evidence)."<br>- Possibly minor improvements (e.g., small BMI reductions in obese women), but overall evidence is very low quality & inconsistent.
Nkrumah et al. 2020	32592533	The Relationship Between Pregnancy Intentions and Diet or Physical Activity Behaviors in the Preconception and Antenatal Periods: A Systematic Review and Meta-Analysis.	19 studies	Exposure: Intended vs. unintended pregnancy; dietary and physical activity behaviors in preconception & antenatal periods	No association between pregnancy intention & preconception fruit/veg or physical activity.<br>- Intended pregnancy correlated with better antenatal diet, lower caffeine, and higher physical activity.

Supplement Table 2. Experimental Studies.

Study	PMID	Title	Sample	Interventions/Exposures	Findings
Van Dijk et al. 2020	32412417	A Mobile App Lifestyle Intervention to Improve Healthy Nutrition in Women Before and During Early Pregnancy: Single-Center Randomized Controlled Trial.	218	Personalized online coaching targeting vegetable, fruit, and folic acid intake	After 24 weeks, reduction in dietary risk score was significantly larger in the intervention group (β=0.75, 95% CI 0.18–1.34), mainly due to increased vegetable intake.
Den Harink et al. 2023	36624285	A preconception lifestyle intervention in women with obesity and cardiovascular health in their children.	49 children (follow-up of original 243)	Preconception lifestyle intervention (diet and activity) for women with obesity, assessing children's cardiac health by MRI.	Offspring of intervention group had a higher left ventricular ejection fraction (63.0% vs 58.8%, p=0.02) and less abnormal cardiac shape compared to controls.
Lumley et al. 2006	17156466	Aiming to increase birth weight: a randomised trial of pre-pregnancy information, advice and counselling in inner-urban Melbourne.	950 women	Multi-component inter-pregnancy counseling (discussion of social/health/lifestyle issues, pregnancy timing, rubella immunization, etc.)	Birth weight in the intervention arm was actually lower than standard care (−97.4 g), and more adverse outcomes (preterm <32 weeks, birth weight <2000 g) were noted in the intervention group.
Claesson et al. 2018	30193722	Effects of a gestational weight gain restriction program for obese women: Sibling pairs' weight development during the first five years of life.	262 children (index children + younger siblings), mothers in either intervention or control group	Mothers had participated in a GWG restriction program during pregnancy; researchers compared children's weight-for-length/height from 2 months to 5 years of age between intervention and control groups, and among younger siblings	Among the intervention group, index boys had a lower mean BMI at 5 years than their younger sisters (p=0.016). No significant differences were observed between index children and their younger siblings regarding maternal GWG or when compared with national reference data. No clear difference in outcomes was observed between the control group's children and siblings.
Van Elten et al. 2018	30403756	Effects of a preconception lifestyle intervention in obese infertile women on diet and physical activity; A secondary analysis of a randomized controlled trial.	577 obese infertile women (RCT: 290 intervention vs. 287 control)	Six-month lifestyle intervention (dietary counseling, physical activity) prior to infertility treatment vs. prompt infertility treatment	Compared to controls, the intervention group reduced sugary drink intake at 3 months by 0.5 glasses/day (95% CI -0.9; -0.2), savory snack intake at 3 months by 2.4 handful/week (95% CI -3.4; -1.4) and at 6 months by 1.4 handful/week (95% CI -2.6; -0.2), and sweet snack intake at 3 months by 2.2 portions/week (95% CI -3.3; -1.0) and at 12 months by 1.9 portions/week (95% CI -3.5; -0.4). They also increased moderate-to-vigorous physical activity at 3 months by 169 minutes/week (95% CI 6.0; 332.1).

Legro et al. 2022	35041662	Effects of preconception lifestyle intervention in infertile women with obesity: The FIT-PLESE randomized controlled trial.	379 women with obesity (BMI ≥30 kg/m²)	Preconception intensive lifestyle intervention (weight loss target ~7% via meal replacements + Orlistat) vs. a standard exercise intervention (without targeted weight loss), followed by standardized infertility treatment	No difference in healthy live birth incidence (standard 15.2% vs. intensive 12.2%, RR 0.81 [0.48–1.34], p=0.40). Intensive group lost significantly more weight (−6.6%±5.4% vs. −0.3%±3.2%, p<0.001) and showed a decrease in metabolic syndrome incidence (from 52.8% to 32.2%, p=0.003). First-trimester pregnancy loss was higher (though nonsignificant) in the intensive group (33.3% vs. 23.7%).
Bastani et al. 2020	20795423	Impact of preconception health education on health locus of control and self-efficacy in women.	210 women attending premarital clinics in the Islamic Republic of Iran (109 intervention, 101 control)	A short-term health education workshop aimed at improving health locus of control and self-efficacy in physical activity prior to marriage (and potential pregnancy)	Post-intervention, the experimental group showed significantly higher scores for internal health locus of control and self-efficacy compared to controls (p<0.05). A brief educational program was able to empower women's intention to adopt healthier lifestyles.
Dietz et al. 2021	34238659	Improvements in PCOS characteristics and phenotype severity during a randomized controlled lifestyle intervention.	183 women with PCOS (BMI>25 kg/m²), randomized to 1-year interventions (two lifestyle arms vs. usual care)	Three-component lifestyle intervention (cognitive behavioral therapy, diet, exercise) ± SMS support vs. usual care, assessing changes in PCOS features (ovulation, hyperandrogenism, ovarian morphology) and body weight	Biochemical hyperandrogenism was 30.9% lower in one lifestyle arm vs. care as usual (p=0.027). Within-group analyses showed significant improvements in ovulatory dysfunction (−30.5 to −39.8%), biochemical hyperandrogenism (−27.8%), and polycystic ovarian morphology (−14.0%) in lifestyle groups. Weight loss reduced the odds of ovulatory dysfunction and hyperandrogenism.
Van Elten et al. 2019	30621789	Preconception lifestyle intervention reduces long term energy intake in women with obesity and infertility: a randomised controlled trial.	577 obese infertile women in the LIFEstyle study, with ~5.5-year follow-up data (diet/PA/BMI subsets: N=175–179)	A six-month preconception lifestyle intervention (vs. prompt infertility treatment), assessing long-term diet, physical activity, and body mass index (BMI)	At 5.5 years post-intervention, no overall BMI difference (–0.5 kg/m², p=0.56) but the intervention group had lower energy intake (–216 kcal/day, p=0.04). Those who successfully lost weight during the intervention maintained a lower BMI (–3.4 kg/m², p=0.01) and lower energy intake (–301 kcal, p=0.04) at follow-up compared to controls or unsuccessful losers. Macronutrient intake, diet quality, and physical activity were similar across groups.

Rono et al. 2014	24524674	Prevention of gestational diabetes through lifestyle intervention: study design and methods of a Finnish randomized controlled multicenter trial (RADIEL).	728 women at high risk for diabetes (from RADIEL)	Lifestyle counseling (diet + physical activity) delivered before, during, and after pregnancy vs. usual care in primary health care settings to prevent GDM and related conditions	Among participants (79.6% had previous GDM, 59.6% had prepregnancy BMI≥30), mean BMI was ≈30 in nonpregnant enrollees and ≈33 in pregnant enrollees. The trial design includes frequent nurse visits every 3 months pre- and post-pregnancy, focusing on diet, exercise, and metabolic markers. Outcome data are pending at publication, but the study is anticipated to clarify optimal timing and setting of interventions for GDM prevention.
Legro et al. 2015	26401593	Randomized Controlled Trial of Preconception Interventions in Infertile Women With Polycystic Ovary Syndrome.	216 women with PCOS (BMI 27–42 kg/m²), 149 randomized (OCP=49; Lifestyle=50; Combined=50)	16-week preconception intervention: continuous OCP vs. lifestyle (diet + exercise + weight loss medication), or both, followed by clomiphene ovulation induction	Lifestyle and Combined groups lost ~6.2–6.4% of baseline weight vs. minimal change in OCP group. Metabolic syndrome prevalence rose in the OCP arm (OR=2.47) but not in Lifestyle or Combined arms. Post-intervention ovulation rates were higher with Lifestyle (60%) and Combined (67%) vs. OCP (46%). Live births were 12% (OCP), 26% (Lifestyle), 24% (Combined) (p=0.13).
Poels et al. 2018	29377741	The effect of a local promotional campaign on preconceptional lifestyle changes and the use of PCC.	540 women total (283 pre-intervention, 257 post-intervention)	Dual-track promotional campaign (for couples + PCC providers) targeting preconception health and PCC uptake	Women exposed to the campaign were more likely to make ≥1 lifestyle change (aOR 1.56; 95% CI 1.02–2.39), specifically reducing/quitting alcohol (aOR 1.72; 95% CI 1.05–2.83). PCC usage (information seeking or HCP consultation) trended higher but did not reach significance.
Ghasemi et al. 2018	28641469	The effect of counselling on preconception lifestyle and awareness in Iranian women contemplating pregnancy: a randomized control trial.	152 women (18–35 y) planning to conceive within 1 year (76 intervention, 76 control)	Six group counseling sessions (twice/week for 3 weeks) on preconception lifestyle vs. usual care	The mean score of preconception lifestyle (adjusted mean difference = 0.17; 95% confidence interval = 0.13-0.21) and awareness about preconception lifestyle (7.8; 8.7-6.9) showed statistically significant improvements in the intervention group compared to the control group.
Elsinga et al. 2008	19059545	The effect of preconception counselling on lifestyle and other behaviour before and during pregnancy.	211 women with PCC vs. 422 matched controls	Preconception counseling vs. standard care, assessing knowledge on pregnancy-related risks, preventive behaviors, and adverse outcomes	PCC participants had higher pregnancy-related knowledge (81.5% vs. 76.9% correct) and were more likely to initiate folic acid pre-pregnancy (aOR=4.93; CI 2.81–8.66) and reduce alcohol in first trimester (aOR=1.79; CI 1.08–2.97). Adverse outcomes were 16% with PCC vs. 20% with standard care (OR=0.77; CI 0.48–1.22).

Dokras et al. 2016	27253669	Weight Loss and Lowering Androgens Predict Improvements in Health-Related Quality of Life in Women With PCOS.	216 overweight/obese women with PCOS (age 18–40; BMI 27–42 kg/m²), 149 analyzed	16-week preconception treatments: oral contraceptive pill (OCP), intensive lifestyle (weight loss + exercise), or combined, assessing effects on health-related quality of life, depression, and anxiety	All arms improved general health (SF-36). OCP and Combined improved all PCOSQ domains ($p<0.01$). Combined had greater improvements vs. single treatments in the weight, body hair, and infertility domains ($p<0.05$). Linear regressions showed that weight reduction correlated with improved weight and physical well-being scores, while decreased testosterone correlated with hair domain improvement. Both contributed to better infertility and menstrual domains.

Supplement Table 3. Observational studies focused on specific prepregnancy lifestyle interventions.

Study	PMID	Title	Study Type	Sample	Interventions/Exposures	Findings
Guendelman et al. 2013	22782493	Association between preterm delivery and pre-pregnancy body mass (BMI), exercise and sleep during pregnancy among working women in Southern California.	Case-control	344 preterm cases and 698 term controls (1042 total)	Pre-pregnancy BMI, second-trimester exercise, and sleep patterns	Moderate second-trimester exercise reduced odds of PTD (OR ≈ 0.90 per hour/week; 95% CI 0.84–0.96), especially in women with BMI >24 kg/m².
Thompson et al. 2003	12875798	Periconceptional multivitamin folic acid use, dietary folate, total folate and risk of neural tube defects in South Carolina.	Case-control	179 women with isolated NTD-affected pregnancies, 288 controls (1992–1997)	Preconception dietary folate/multivitamin folic acid intake (≥0.4 mg) in the 3 months before conception and first 3 months of pregnancy, in relation to risk of isolated neural tube defects (NTDs)	Use of ≥0.4 mg folic acid ≥3 times/week was not significantly protective (AOR=0.55; 95% CI 0.25–1.22). However, highest quartiles of dietary folate and total folate (diet + supplements) were associated with reduced NTD risk (AOR=0.40 [95% CI 0.19–0.84], AOR=0.35 [0.17–0.72], respectively).
Agricola et al. 2014	24731520	A cohort study of a tailored web intervention for PCC.	Prospective Cohort	508 enrolled, 282 completed	Tailored web-based preconception health recommendations (folic acid, alcohol, smoking, etc.)	Logistic regression showed that having a preconception visit was significantly associated with increased folic acid use (OR 2.53, 95% CI 1.40–4.60); reductions in alcohol use and improved knowledge.
Wade et al. 2012	22417917	A PCC program for women in a college setting.	Prospective Cohort	~100 college women	Peer-education preconception health program covering multiple risk factors (e.g., REFRAMED PLUS mnemonic)	Pretest vs. posttest showed increased knowledge of preconception health, though no specific statistics (p-values, ORs) reported.
Kumar et al. 2022	35682375	Automated Machine Learning (AutoML)-Derived Preconception Predictive Risk Model to Guide Early Intervention for Gestational Diabetes Mellitus.	Prospective Cohort	222	Building a predictive model for GDM risk using preconception metabolic markers (HbA1c, insulin, lipids)	AutoML model achieved AUC=0.93; each 1 mmol/mol increase in preconception HbA1c raised risk of GDM (OR 1.34) and PTB (OR 1.63).

Sijpkens et al. 2021	32431156	Change in Lifestyle Behaviors After PCC: A Prospective Cohort Study.	Prospective Cohort	259 enrolled (177 self-report, 82 biomarker)	Voluntary PCC (PCC) consultation focusing on folic acid, smoking, alcohol, etc.	Prevalence of no folic acid use decreased (p<0.001) and binge drinking dropped (p=0.007) after PCC; biomarker data also confirmed increased folate status.
Gaskins et al. 2019	30742825	Dietary patterns and outcomes of assisted reproduction.	Prospective Cohort	357 women (608 ART cycles)	Adherence to four dietary patterns: Mediterranean diet, alternate Healthy Eating Index, Fertility Diet, and "pro-fertility diet"	Women in quartiles 2–4 of Mediterranean diet adherence had a higher probability of live birth (0.44, 95% CI: 0.39–0.49) versus quartile 1 (0.31, 95% CI: 0.25–0.39), but no additional benefit was observed above quartile 2. Each SD increase in "pro-fertility diet" adherence increased implantation odds by 47% (95% CI: 21–77%), clinical pregnancy odds by 43% (95% CI: 19–72%), and live birth odds by 53% (95% CI: 26–85%), and reduced clinical pregnancy loss (OR=0.69, 95% CI: 0.53–0.90).
Crozier et al. 2009	19689495	Do women change their health behaviours in pregnancy? Findings from the Southampton Women's Survey.	Prospective Cohort	1,490 women in the Southampton Women's Survey (delivered 1998–2003)	Diet and lifestyle factors (smoking status, fruit/vegetable intake, alcohol use, and caffeine intake) assessed before pregnancy and at 11 and 34 weeks' gestation	Before pregnancy, 27% smoked vs. 15% in early pregnancy; 54% drank >4 units of alcohol/week pre-pregnancy vs. 10% in early pregnancy; 39% exceeded 300 mg/day caffeine before pregnancy vs. 16% in early pregnancy. Little change was seen in fruit/vegetable intake (47% vs. 46% not meeting "5-a-day"). Younger women and those with fewer educational qualifications were less likely to comply with recommendations. Overall, 81% of women in early pregnancy complied with at least three lifestyle guidelines.
Stanford et al. 2019	31731946	Fecundability in relation to use of fertility awareness indicators in a North American preconception cohort study.	Prospective Cohort	5,688 North American women (web-based cohort)	Use of fertility awareness indicators (e.g., charting cycles, basal body temperature, cervical fluid monitoring, LH tests) in women attempting conception	At baseline, 75% used ≥1 fertility awareness method; adjusted fecundability ratio (FR) for any indicator use vs. none = 1.25 (95% CI 1.16–1.35). Individual indicators had FRs ranging 1.28–1.36, and using a combination of charting days, cervical fluid, and LH tests had FR=1.48 (1.31–1.67).
Van Dijk et al. 2017	28688924	Healthy preconception nutrition and lifestyle using personalized mobile health coaching is associated with enhanced pregnancy chance.	Prospective Cohort	1,053 women and 332 male partners (total n=1,385)	Personalized mHealth coaching ("Smarter Pregnancy") for 26 weeks addressing poor nutrition and lifestyle, assessing subsequent chance of pregnancy	A higher total risk score (reflecting more poor behaviors) was significantly associated with a lower chance of pregnancy (aHR 0.79; 95% CI 0.72–0.85), especially when the male partner also participated (aHR 0.75; 95% CI 0.61–0.91). Associations held for both infertile and fertile couples.

Ockhuijsen et al. 2012	22032346	Integrating preconceptional care into an IVF programme.	Prospective Cohort	130 couples (101 patients responded), plus 7 nurses	Integration of preconceptional care into an IVF program (focus on lifestyle modification, smoking cessation, weight reduction)	Nurses were initially skeptical, but patients appreciated lifestyle support to improve fertility. Among those with BMI>30 or who smoked, 30% quit smoking (7/23) and 50% lost weight (15/30), with a mean loss of 6.1 kg.
Livock et al. 2017	27485466	Maternal micronutrient consumption periconceptionally and during pregnancy: a prospective cohort study.	Prospective Cohort	2,146 Australian pregnant women	Periconceptional and pregnancy micronutrient intake (from diet + supplements), plus sociodemographic/lifestyle correlates	Women with planned pregnancies, older age (>25), Caucasian background, higher education, non-smoking status, and folate-rich diets were more likely to use supplements. Across pregnancy, 19–46% did not meet RDI for folate, 68–82% for iron, and 17–36% for zinc; 15–19% exceeded the Upper Limit for folate and 11–24% for iron. Micronutrient intakes were significantly lower in the periconceptional period than later in pregnancy.
Gootjes et al. 2019	30973345	Neighborhood Deprivation and the Effectiveness of Mobile Health Coaching to Improve Periconceptional Nutrition and Lifestyle in Women: Survey in a Large Urban Municipality in the Netherlands.	Prospective Cohort	2,554 women in the "Smarter Pregnancy" mHealth program (2011–2016)	24-week lifestyle/nutrition coaching, comparing effects by neighborhood deprivation status	Women living in more deprived neighborhoods were more likely to complete the program and showed greater improvements in inadequate lifestyle factors (OR=0.89 [0.82–0.97] for vegetable intake improvement). Overall, 26–64% achieved better diet/lifestyle after 24 weeks, but unexpectedly, those from non-deprived areas improved less (e.g., they smoked less but had lower program adherence).
Grieger et al. 2019	31404968	Pre-Conception Maternal Food Intake and the Association with Childhood Allergies.	Prospective Cohort	234 mother–child pairs	Maternal preconception food frequency intake in relation to childhood eczema, wheeze, and rhinitis up to age 3	Linear discriminant analysis suggested certain foods (e.g., low/high fat dairy, fresh fruit, non-oily fish, saturated spreads) were protective against child allergy, whereas others (e.g., poultry, fruit juice) were adversely associated. Results were inconsistent and sometimes contradictory, indicating complexity in how maternal diet might influence fetal programming of allergic disorders.
Siega-Riz et al. 2021	33187928	Preconception Diet Quality Is Associated with Birth Weight for Gestational Age Among Women in the Hispanic Community Health Study/Study of Latinos.	Prospective Cohort	497 Hispanic/Latina mother–infant dyads in the Hispanic Community Health Study/Study of Latinos	Overall diet quality (HEI-2010) in the preconception period and infant birth weight (adjusted for gestational age)	In adjusted models, women in the highest tertile of the HEI-2010 had infants with higher birth-weight-for-gestational-age percentiles than those in the lowest tertile. A continuous higher HEI-2010 score also correlated with better birth weight outcomes, regardless of maternal preconception BMI.

Van Elten et al. 2019	31615021	Preconception Lifestyle and Cardiovascular Health in the Offspring of Overweight and Obese Women.	Prospective Cohort	46 mother–child pairs from a Dutch preconception lifestyle intervention study	Women's preconception diet and physical activity (over 6 months) and offspring cardiovascular measures (BMI, BP, pulse wave velocity) at 3–6 years old	Each 10 g/day increase in preconception vegetable intake was associated with lower offspring diastolic BP Z-score (–0.05; p=0.007). Each 10 g/day fruit intake was linked to lower pulse wave velocity (–0.05 m/s; p=0.03). Conversely, higher sugary drink intake correlated with higher child fat-free mass.
Lyden et al. 2020	32249967	Pregnancy intention and phthalate metabolites among pregnant women in The Infant Development and Environment Study cohort.	Prospective Cohort	721 pregnant women in the TIDES multicenter US cohort	Pregnancy intention (planned vs. unplanned) in relation to phthalate metabolite concentrations (10 biomarkers) in first and third trimesters	In unadjusted analyses, unplanned pregnancies had higher geometric mean phthalate metabolite concentrations in first and most third-trimester measures. However, after adjusting for demographics and socioeconomic factors, only first-trimester MiBP remained significantly different (with unplanned pregnancies showing lower levels).
Li et al. 2019	31010874	Prepregnancy Habitual Intakes of Total, Supplemental, and Food Folate and Risk of Mellitus: A Prospective Cohort Study.	Prospective Cohort	14,553 women from the Nurses' Health Study II (824 GDM cases)	Prepregnancy folate intake (total, supplemental, dietary) in relation to incident mellitus (GDM)	Women meeting ≥400 µg/day total folate had a 17% lower GDM risk (RR=0.83; 95% CI 0.72–0.95). Effects were driven by supplemental folate (≥600 µg/day vs. none = RR=0.70; 95% CI 0.54–0.91). The association persisted after adjusting for other micronutrients and was particularly evident among women who were likely planning pregnancy.
Hammiche et al. 2011	21752799	Tailored preconceptional dietary and lifestyle counselling in a tertiary outpatient clinic in The Netherlands.	Prospective Cohort	419 subfertile couples receiving dietary/lifestyle counseling, with a 3-month follow-up in 110 couples	Tailored preconception counseling for dietary/lifestyle factors (e.g., fruit/veg intake, alcohol, exercise), with repeated assessment over ~3 months	After counseling, women's fruit consumption rose from 65% to 80% meeting guidelines, and men's from 49% to 68%. Women's fish intake rose 39%→52%. Alcohol use dropped 14.6% in women and 19.4% in men; physical activity increased, and folic acid use improved. Median diet and lifestyle risk scores both decreased significantly in all SES and BMI categories. Over 85% deemed the counseling useful, with ~70% recommending it.
Akhter et al. 2016	27623482	The impact of periconceptional maternal stress on fecundability.	Prospective Cohort	400 women (139 conceptions)	Daily self-reported stress (scale 1–4) during the follicular/ovulatory and luteal phases in up to 20 cycles or until pregnancy	A 1-unit increase in follicular-phase stress (particularly during ovulatory window) was associated with a 46% lower fecundability (FOR=0.54; 95% CI 0.35–0.84). In the luteal phase, higher stress coincided with higher likelihood of conception (FOR=1.63), possibly due to reverse causality (awareness of pregnancy).
Inskip et al. 2009	19213768	Women's compliance with nutrition and lifestyle recommendations before	Prospective Cohort	12,445 UK women (20–34 y) in the Southampton	Nutrition and lifestyle factors (folic acid, alcohol, smoking, fruit/vegetables,	Among those who conceived, only 2.9% met both folic acid ≥400 µg/day & ≤4 alcohol units/week. 74% were nonsmokers vs. 69% in non-pregnant group (p=0.08). 53% in both groups consumed ≥5 fruit/veg/day. Only 57% of the

		pregnancy: general population cohort study.		Women's Survey (SWS); 238 became pregnant within 3 months	exercise) in nonpregnant women vs. those who conceived within 3 months	conceiving group exercised strenuously vs. 64% in non-pregnant (p=0.03). Overall compliance with preconception guidelines was low.
Lum et al. 2011	21658667	Women's lifestyle behaviors while trying to become pregnant: evidence supporting preconception guidance.	Prospective Cohort	90 women followed from contraception discontinuation until pregnancy (459 cycles)	Daily self-reported cigarette, alcohol, and caffeine use while attempting pregnancy, along with women's self-stated intention to change these behaviors	Despite an overall drop in caffeine use (–0.52 drinks/day, p<0.001), alcohol and smoking rates did not show a statistically significant decrease unless women explicitly intended to change. Intention was strongly associated with reduced cigarettes (EST=–1.65) and alcohol (EST=–0.15) from cycle 1 onward.
Davidson et al. 2020	33355674	Association of Improved Periconception Hemoglobin A1c With Pregnancy Outcomes in Women With Diabetes.	Retrospective Cohort	3459 births among women with pre-pregnancy diabetes	Net decline in HbA1c from preconception to mid-pregnancy (glycemic control in diabetic women)	Each 0.5% net decrease in HbA1c was linked to a lower risk of congenital anomalies (aRR=0.94), PTB (aRR=0.89), and severe maternal morbidity (aRR=0.90).
Donovan et al. 2019	30978258	Development and validation of a clinical model for preconception and early pregnancy risk prediction of mellitus in nulliparous women.	Retrospective Cohort	Large California birth cohort (n not stated) + external Iowa cohort (2009–2017)	Five risk factors: race/ethnicity, age at delivery, pre-pregnancy BMI, family history of diabetes, and pre-existing hypertension	A risk prediction model was developed and validated. The California internal validation showed an AUC of 0.732 (95% CI: 0.728–0.735). The Iowa external validation showed an AUC of 0.710 (95% CI: 0.672–0.749). The model performed particularly well for Hispanic (AUC=0.739) and Black women (AUC=0.719). Women with predicted risk >6% might benefit most from preventive intervention.
Witt et al. 2016	26767530	Infant birthweight in the US: the role of preconception stressful life events and substance use.	Retrospective Cohort	9,350 women-child dyads from the Early Childhood Longitudinal Study-Birth Cohort	Preconception stressful life events (PSLEs), tobacco and alcohol use in relation to birthweight	PSLEs increased the odds of very low birthweight (VLBW) by 35% (AOR=1.35; 95% CI 1.10–1.66). Smoking prior to conception (AOR=1.31; 95% CI 1.04–1.66) and in the last trimester (AOR=1.98; 95% CI 1.56–2.52) also elevated risk of low birthweight (LBW).
Oostingh et al. 2019	31647476	Mobile Health Coaching on Nutrition and Lifestyle Behaviors for Subfertile Couples Using the Smarter Pregnancy Program: Model-Based Cost-Effectiveness Analysis.	Retrospective Cohort	793 subfertile women undergoing IVF at Erasmus MC, the Netherlands	"Smarter Pregnancy" mHealth coaching program versus usual care for subfertile couples prior to first IVF cycle	The decision tree model estimated 86 additional pregnancies and ~€270,000 saved after two IVF cycles with mHealth (ICER=–€3050 [95% CI –3960 to –540] per additional pregnancy). Largest cost savings stemmed from avoided IVF treatment costs. Sensitivity analysis showed the program must increase ongoing pregnancy rates by at least 51% to remain cost-saving.

Nobles et al. 2018	29610265	Preconception Blood Pressure Levels and Reproductive Outcomes in a Prospective Cohort of Women Attempting Pregnancy.	Retrospective Cohort	1,228 women from the EAGeR trial (history of pregnancy loss)	Preconception blood pressure (BP) (systolic, diastolic, mean arterial pressure) in relation to fecundability, pregnancy loss, and live birth	In adjusted models, each 10 mmHg increase in diastolic BP was associated with an 18% higher risk of pregnancy loss (95% CI 1.03–1.36), and each 10 mmHg increase in mean arterial pressure was associated with a 17% higher risk (95% CI 1.02–1.35). No associations were found with fecundability or live birth rates.
Wang et al. 2021	34627684	Preconception insulin resistance and neonatal birth weight in women with obesity: role of bile acids.	Retrospective Cohort	469 women with obesity (BMI ≥29 kg/m²) in the LIFEstyle RCT, 238 singleton births analyzed	Preconception insulin resistance (HOMA-IR) and bile acid levels, in relation to neonatal birth weight	Higher HOMA-IR was associated with higher total bile acids preconception (adjusted B=0.15; 95% CI 0.09–0.22). HOMA-IR before pregnancy was positively linked with birth weight Z-score (B=0.08; p=0.03), while no association was found between bile acids and birth weight.
Witt et al. 2015	25449635	Predictors of alcohol and tobacco use prior to and during pregnancy in the US: the role of maternal stressors.	Retrospective Cohort	9,350 women-child dyads from Early Childhood Longitudinal Study-Birth Cohort	Preconception stressful life events (PSLEs) and women's alcohol/tobacco use before and during pregnancy	Women with PSLEs had increased odds of tobacco use preconception (AOR=1.52) and during pregnancy (AOR=1.57). They smoked ~5 more packs in the 3 months before pregnancy (p=0.011) and drank 0.31 more alcoholic drinks in the last 3 months than unexposed women.
Ritchie et al. 2023	36943523	Reducing Maternal Obesity and Diabetes Risks Prior to Conception with the National Diabetes Prevention Program.	Retrospective Cohort	1,569 participants in National Diabetes Prevention Program; analysis of 32 who became pregnant vs. 26 controls	NDPP (1-year lifestyle program) delivered in a safety net system to diverse, low-income women; outcomes compared with usual care for preconception BMI and hyperglycemia	Women in NDPP lowered BMI from baseline to conception by −1.8 kg/m² vs. usual care (p=0.002). NDPP participants were less likely to have obesity at conception (57% vs. 88%) or early pregnancy hyperglycemia (4% vs. 25%). Adjusted models remained similar, though the difference in obesity prevalence at conception was no longer significant.
Ding et al. 2015	26249750	Survey on the Implementation of PCC in Shanghai, China.	Retrospective Cohort	12,309 pregnant women + 8,997 partners in Shanghai (multi-hospital study)	Government-led PCC (PCC) in Shanghai, assessing uptake, lifestyle changes, and demographics	40% of women and 35% of men had used PCC services; participants were more likely to have planned pregnancies and higher education/income. PCC use was linked to healthier behaviors preconception (e.g., folic acid use, smoking cessation, reduced alcohol/drug exposure). Future efforts should target younger, less educated, low-income couples.
Mumford et al. 2014	23797269	Women's longitudinal smoking patterns from preconception through child's kindergarten	Retrospective Cohort	8,650 biological mothers from the Early Childhood	Smoking trajectories from preconception through early parenting,	Five patterns were identified: 71.7% nonsmokers, 4.3% pregnancy-inspired quitters, 5.1% delayed initiators, 8.5% persistent smokers, 10.4% temporary quitters. Predictors included lower education (≥5× more likely to be in

		entry: profiles of biological mothers of a 2001 US birth cohort.		Longitudinal Study-Birth Cohort (US)	identifying latent classes: nonsmokers, pregnancy-inspired quitters, delayed initiators, persistent smokers, temporary quitters	delayed/persistent/temporary than nonsmoker). Other correlates: marital status, poverty, parity, alcohol, depression.
Poels et al. 2017	28478375	Actively preparing for pregnancy is associated with healthier lifestyle of women during the preconception period.	Cross-sectional	283	Actively seeking pregnancy information or consulting a healthcare provider vs. not preparing	Adjusted ORs for improved behaviors: diet (7.84, 95% CI 3.03–20.30), folic acid use (3.90, 95% CI 2.00–7.62), alcohol cessation (5.46, 95% CI 1.76–16.96).
Dennis et al. 2023	34809524	Describing 24-hour movement behaviours among preconception and recently pregnant Canadian parents: who do we need to target?	Cross-sectional	1304 Canadian adults (1080 F, 224 M)	Self-reported physical activity, sedentary time, sleep, and screen time	54.0% of participants met the physical activity guideline; 78.4% met the sedentary behavior guideline; 56.4% met the sleep guideline; and 15.4% met the screen time guideline. Only 5.0% (n=60) met all four guidelines. Multiple logistic regressions indicated higher odds of meeting more guidelines with parity and perceived health, and lower odds with overweight/obesity and depression (no specific OR/CI values reported).
Vink-van et al. 2015	25967756	Determining Pre-Conception Risk Profiles Using a National Online Self-Reported Risk Assessment: A Cross-Sectional Study.	Cross-sectional	66617 non-pregnant women	Lifestyle, medical, reproductive, and family history risk factors	Chi-square (χ^2) tests were used to compare risk profiles among sociodemographic subgroups. Women older than 36 years, of non-Western origin, living in urban areas, and those in deprived neighborhoods had significantly higher prevalences of risk factors (exact percentages not provided). Overall, a substantial number of risk factors were identified across the population.
Cuervo et al. 2014	25333199	Dietary and health profiles of Spanish women in preconception, pregnancy and lactation.	Cross-sectional	12845 women	Dietary patterns and lifestyle factors (preconception, pregnancy, and lactation)	Mean values, percentages, and group comparisons were performed (t-test, ANOVA, chi-square). Women seeking pregnancy only met dairy recommendations; pregnant women only fulfilled fruit recommendations; lactating women only met protein group requirements. In all groups, the consumption of sausages, buns, and pastries exceeded recommendations.
Ahmed et al. 2020	32850148	Does the Frequency of Watching Television Matters on Overweight and Obesity among Reproductive Age Women in Ethiopia?	Cross-sectional	10,074 reproductive-age women from the 2016 Ethiopian Demographic and Health Survey	Frequency of watching television as the main exposure, with overweight/obesity as outcomes	Watching television at least once a week was associated with higher odds of being overweight (AOR 1.79; 95% CI 1.20–2.73) and obese (AOR 3.76; 95% CI 2.04–6.95). The odds of overweight also increased with age (25–39 years AOR 2.17; 95% CI 1.25–3.77; 40–49 years AOR 2.69; 95% CI 1.45–5.00), urban residence (AOR 1.76; 95% CI 1.17–2.65), higher education (AOR 2.11; 95%

						CI 1.22–3.65), and highest wealth index (AOR 2.83; 95% CI 1.71–4.68). Similar patterns were observed for obesity.
Xaverius et al. 2009	19105688	Exploring health by reproductive status: an epidemiological analysis of preconception health.	Cross-sectional	66,152 fertile women aged 18–44 from BRFSS (2002 & 2004)	Pregnancy status and intention (pregnant, intending pregnancy, or varying risk groups) related to health behaviors (alcohol use, smoking, folic acid use, leisure activity)	Women who were not currently pregnant (IP, HR, MR, LR groups) were much more likely than pregnant women to consume alcohol (any, binge, heavy) and to smoke, but also more likely to engage in leisure activity. HR, MR, and LR groups were significantly less likely to take folic acid, while women intending pregnancy were equally likely to take it (though data had low response rates).
Hultstrand et al. 2020	31914332	Foreign-born women's lifestyle and health before and during early pregnancy in Sweden.	Cross-sectional	3,389 women in Sweden (414 foreign-born)	Comparison of lifestyle/health before and during early pregnancy between Nordic-born vs. foreign-born women (European vs. non-European), including alcohol, tobacco, physical activity, BMI, and mental health	Foreign-born women consumed less alcohol preconception (European-born aOR=0.38; 95% CI 0.24–0.58, non-European aOR=0.14; 95% CI 0.10–0.19) and in early pregnancy (European-born aOR=0.61; 95% CI 0.40–0.91, non-European aOR=0.20; 95% CI 0.14–0.29). Non-European women also used less tobacco but were less physically active; they showed a higher prevalence of depressive symptoms (aOR=1.67; 95% CI 1.12–2.51). Religiousness was associated with healthier behaviors.
Mwase-Musicha et al. 2022	35286337	How do women prepare for pregnancy in a low-income setting? Prevalence and associated factors.	Cross-sectional	4,244 pregnant mothers recruited in Mchinji district, Malawi	Forms of pregnancy preparation (e.g., eating more healthily, saving money) and associations with socio-demographic and obstetric factors	36.1% of women took some action to prepare for pregnancy (most commonly healthier eating [71.9%] and saving money [42.8%]). Women who were married (AOR=7.77; 95% CI 5.31–11.25), had fewer living children, or had ≥2-year birth intervals were more likely to prepare, whereas teenage (<20 y) and older (≥35 y) mothers were less likely (AOR=0.61 and 0.49, respectively).
Lang et al. 2021	32312651	Opportunities for enhancing pregnancy planning and preconception health behaviours of Australian women.	Cross-sectional	317 pregnant women (>18 y) in Australia (public maternity service=225, national private insurer=92)	Associations between pregnancy planning status, sociodemographics, and preconception health behaviors (folic acid use, weight management, immunizations, smoking, health care engagement)	74% of pregnancies were planned. Planned pregnancy was associated with being married (AOR=5.71; 95% CI 1.92–17.00), having ≤2 children (AOR=3.75; 95% CI 1.28–11.05), and having private insurance (AOR=2.51; 95% CI 1.08–5.81). Planners were more likely to use folic acid (AOR=17.13), review immunizations (AOR=2.09), and seek information (AOR=3.24). Women <25 y were less likely to take folic acid or seek information and more likely to smoke preconception.

Maas et al. 2022	35854217	Planning is not equivalent to preparing, how Dutch women perceive their pregnancy planning in relation to preconceptional lifestyle behaviour change - a cross-sectional study.	Cross-sectional	1,077 low-risk pregnant women in the APROPOS-II study, the Netherlands	Self-reported preconceptional behaviors (diet, smoking, alcohol, folic acid, exercise) and planning status (London Measure of Unplanned Pregnancy), plus beliefs about health and PCC	85.5% had a planned pregnancy, but only ~69.5% used folic acid correctly. Half (50.5%) still drank alcohol, and 30.4% were under-/overweight despite believing they were "healthy enough." Many women overestimated their health, interfering with active preparation. Multivariate analysis showed that women's health beliefs did not consistently translate into healthy preconception behaviors.
Bombard et al. 2013	23286663	PCC: the perfect opportunity for health care providers to advise lifestyle changes for hypertensive women.	Cross-sectional	2,063 reproductive-age women (18–44 y) with self-reported hypertension from BRFSS (2009)	Health care provider lifestyle advice (on diet, salt reduction, exercise, alcohol) and women's subsequent self-reported behavior changes	Most women reported receiving advice about diet (73%), salt (75%), and exercise (82%), but only 45% were advised to reduce alcohol. Women who received such advice were significantly more likely to adopt the recommended behavior changes (PRs ranged ~1.3–1.6).
Deierlein et al. 2022	35041530	Preconception Health and Disability Status Among Women of Reproductive Age Participating in the National Health and Nutrition Examination Surveys, 2013-2018.	Cross-sectional	4,055 women of reproductive age (18-44 y) in NHANES 2013-2018	Disability status (self-reported) and preconception health indicators (education, income, smoking, BMI, multivitamin use, etc.)	15% reported ≥1 disability, with higher rates of suboptimal health indicators (aPR range ~1.1–2.0) compared to women without disabilities. The greatest disparities were in fair/poor general health, depression, and diabetes (aPRs 2.4–3.8).
Bish et al. 2012	23099798	Preconception health of reproductive aged women of the Mississippi River delta.	Cross-sectional	171,612 nonpregnant Black and White women (18-44 y) from the BRFSS (2005–2009)	Region of residence (MS Delta vs. other areas) in relation to 16 preconception health indicators (e.g., fruit/veg intake, BMI, physical activity)	Compared to other regions, the Mississippi Delta had lower prevalence of healthy PCH factors, such as adequate produce intake (aPR=1.7 comparing non-Delta states vs. Delta) and normal BMI (aPR=1.4). Race and household income confounded these relationships.
Goossens et al. 2018	29096279	Preconception lifestyle changes in women with planned pregnancies.	Cross-sectional	430 women with a planned pregnancy, 6 Flemish hospitals (Belgium)	Preconception lifestyle changes (folic acid intake, smoking/alcohol/caffeine changes, healthy weight, medical advice, etc.) and their associated factors	83% reported ≥1 lifestyle change preconception. Nulliparous women (OR=2.18) and those with previous miscarriage (OR=2.44) were more likely to prepare, whereas lower education (OR=0.56) and financial struggles (OR=0.20) reduced likelihood. Nearly half received formal preconception advice; 77% of those who made no changes had ≥1 risk factor for adverse outcomes.

Goossens et al. 2016	26564478	Preconception-related needs of reproductive-aged women.	Cross-sectional	242 reproductive-age women in Belgium with childbearing intentions	Women's interest in receiving PCC (PCC), preferred providers (gynecologists, midwives, GPs), and specific information/support needs (e.g., lifestyle, exposures, medical issues)	75% wanted PCC in the future. Gynecologists were most preferred (93%), followed by midwives (73%) and GPs (63%). Women with a history of mental illness or overweight reported higher info/support needs, especially regarding lifestyle. Although midwives are less often sought, authors suggest they can have a larger role with adequate training in PCC.
Kazemi et al. 2020	32631153	Using social cognitive theory to explain physical activity in Iranian women preparing for pregnancy.	Cross-sectional	220 women (Iran) in PCC settings	Physical activity level (moderate/vigorous) measured by IPAQ, plus social cognitive theory (SCT) constructs (observational learning, self-efficacy, outcome expectations, etc.)	Structural equation modeling showed observational learning (β=0.65) as the strongest predictor of physical activity levels. Model fit was acceptable (CMIN/DF=2.10, p=0.097). Other SCT constructs contributed but observational learning had the largest effect.
Pandolfi et al. 2014	24885235	Women participating in a web-based preconception study have a high prevalence of risk factors for adverse pregnancy outcomes.	Cross-sectional	728 Italian women enrolled via a web platform (mammainforma.it)	Preconception risk factors for adverse pregnancy outcomes (BMI, smoking, alcohol, folic acid use, rubella/hepatitis susceptibility, etc.)	70% used alcohol, 16% smoked, 6% underweight, 21% overweight, 51.6% no folic acid, 22% rubella-susceptible. Nulliparous women had higher BMI risk (OR=1.60) but lower odds of smoking or drinking. Knowledge about risk factors correlated with corrective behaviors (e.g., lower smoking/drinking, folic acid use).

Supplement Table 4. Observational studies focused on the importance of prepregnancy lifestyle modification.

Study	PMID	Title	Study Type	Sample	Interventions/Exposures	Findings
Asadi et al. 2019	30729640	The relationship between pre-pregnancy dietary patterns adherence and risk of mellitus in Iran: A case-control study.	Case-control	278 pregnant women (Iran), 6 healthcare centers	Pre-pregnancy/prenatal adherence to two major dietary patterns (Western vs. prudent), determined by principal component analysis, and risk of	The prudent dietary pattern (fruits, low-fat dairy, poultry, fish, nuts, etc.) was linked to a reduced GDM risk (OR=0.88; 95% CI 0.44–0.99). No significant association was found between the Western pattern and GDM.

Maas et al. 2021	34233654	Associations between periconceptional lifestyle behaviours and adverse pregnancy outcomes.	Prospective Cohort	3684 pregnant women	BMI, smoking, and folic acid use in the periconception vs. first trimester	Overweight (aOR=1.61) or obesity (aOR=2.85) raised odds of adverse outcomes; continuing smoking increased risk of SGA (aOR=1.91), while preconceptional folic acid use lowered risk.
Bernard et al. 2019	30888073	Associations of physical activity levels and screen time with oral glucose tolerance test profiles in Singaporean women of reproductive age actively trying to conceive: the S-PRESTO study.	Prospective Cohort	946 women	Moderate and vigorous physical activity, screen time as sedentary behavior	Women engaging in ≥75 min/week of vigorous activity had significantly lower fasting (−0.14 mmol/L), 30-min (−0.35 mmol/L), and 120-min (−0.53 mmol/L) glucose levels vs. non-vigorous group; no effect from screen time.
Loy et al. 2021	34172036	Fecundability in reproductive aged women at risk of sexual dysfunction and associated risk factors: a prospective preconception cohort study.	Prospective Cohort	513 preconception Asian women (Singapore S-PRESTO cohort)	Female sexual function (FSF) assessed by FSFI-6, plus lifestyle/behavior (physical activity, obesity, depression/anxiety) in relation to time-to-pregnancy	58.9% had “low FSF” (FSFI-6 ≤22). Low FSF was associated with a 27% reduction in fecundability (FR=0.73; 95% CI 0.54–0.99) compared to high FSF, adjusted for demographic factors. Physical activity, obesity, and absence of depression/anxiety were linked to higher FSF.
Zhu et al. 2021	33900396	Healthy preconception and early-pregnancy lifestyle and risk of PTB: a prospective cohort study.	Prospective Cohort	2,449 pregnant women (singleton pregnancies)	Preconceptional/early pregnancy modifiable factors (healthy weight, high-quality diet, low-to-moderate stress) and the risk of PTB	PTB occurred in 6.5% (n=160). Having a healthy weight, high-quality diet, or low-to-moderate stress each lowered PTB risk (aRR range ~0.58–0.68). Women with 3 low-risk factors vs. none had 70% lower PTB risk (aRR=0.30; 95% CI 0.13–0.70). Associations were stronger for medically indicated vs. spontaneous PTB and late vs. earlier PTB, with some variation by race/ethnicity.
Salih et al. 2019	31419245	Investigating the effect of lifestyle risk factors upon number of aspirated and mature oocytes in in vitro fertilization cycles: Interaction with antral follicle count.	Prospective Cohort	674 women across two Swedish IVF cohorts (242 in “Lifestyle study,” 432 in “UppSTART”)	Smoking, high BMI, and their cumulative impact on IVF outcomes (number of oocytes, maturity rate)	Women who both smoked and had a high BMI showed a significantly lower number of aspirated oocytes (IRR=0.75; 95% CI 0.61–0.94). An interaction was found between BMI and antral follicle count (AFC) for mature oocytes: lower AFC magnified BMI’s negative impact.
Dhana et al. 2018	29568108	Lifestyle of women before pregnancy and the risk of offspring obesity during	Prospective Cohort	5,701 children (ages 9–14 at baseline) from the	Mothers’ pre-pregnancy healthy lifestyle factors (normal BMI, no smoking,	Offspring obesity risk was 63% lower for mothers with healthy weight (RR=0.37; 95% CI 0.31–0.43) and 36% lower for nonsmoking mothers

		childhood through early adulthood.		Growing Up Today Study 2 + maternal data (Nurses' Health Study II)	≥150 min/week physical activity, healthier diet) and offspring obesity risk	(RR=0.64; 95% CI 0.49–0.84). Adherence to all 4 healthy lifestyle factors reduced children’s obesity risk by 75% (RR=0.25; 95% CI 0.14–0.43).
Salavati et al. 2020	33081304	Offspring Birth Weight Is Associated with Specific Preconception Maternal Food Group Intake: Data from a Linked Population-Based Birth Cohort.	Prospective Cohort	1,698 Dutch women from the Perined-Lifelines linked birth cohort	Preconception intake of specific food groups (from a semi-quantitative food frequency questionnaire) in relation to term birth weight	Linear regression adjusting for energy intake and covariates showed that higher intake of “artificially sweetened products” (β=0.001 per 10g/1000 kcal; p=0.002) and “vegetables” (β=0.002; p=0.03) correlated with higher birth weight z-scores. “Egg” intake correlated with lower birth weight (β=–0.093; 95% CI –0.174 to –0.013, p=0.02). Authors highlight the need to further investigate artificial sweeteners’ impact from preconception onwards.
De Lima et al. 2016	27001269	PCC Reduces Relapse of Inflammatory Bowel Disease During Pregnancy.	Prospective Cohort	317 women with inflammatory bowel disease (IBD): 155 in the PCC group, 162 in the no-PCC group	PCC (PCC) in women with IBD vs. no PCC, measuring medication adherence, smoking cessation, disease relapse, and birth outcomes	Adjusted analyses showed that PCC was associated with greater adherence to IBD medication (aOR=5.69), better folic acid intake (5.26), and more smoking cessation (4.63). PCC also halved the odds of disease relapse during pregnancy (aOR=0.51) and strongly reduced the risk of low birth weight infants (aOR=0.08).
Baptiste-Roberts et al. 2011	21951267	Pregravid physical activity, dietary intake, and glucose intolerance during pregnancy.	Prospective Cohort	152 pregnant women in the Parity, Inflammation, and Diabetes (PID) study	Prepregnancy dietary intake (using a Block Rapid Food Screener) and leisure-time physical activity (Baecke questionnaire) in relation to 1-hour glucose challenge test (GCT) at 26–28 weeks	Women with higher prepregnancy leisure activity scores were 68% less likely to have an elevated GCT (≥140 mg/dL). No significant association was observed between prepregnancy dietary intake and GCT result.
Kyozuka et al. 2021	33545538	Prepregnancy antiinflammatory diet in pregnant women with endometriosis: The Japan Environment and Children's Study.	Prospective Cohort	Large subset of the Japan Environment and Children’s Study (n=88,393)	Prepregnancy dietary inflammatory index (DII) in women with endometriosis, evaluating risk of PTB and low birth weight (LBW)	Among women not undergoing fertility treatment, those in the highest (most antiinflammatory) DII quintile had significantly reduced odds of PTB <34 wks (aOR=0.25; 95% CI 0.07–0.83) and LBW <1500 g (aOR=0.07; 95% CI 0.01–0.60). The antiinflammatory diet did not show a clear effect on moderate PTB or LBW thresholds.

De et al. 2021	33368141	Degree of pregnancy planning and recommended pregnancy planning behavior among women with and without chronic medical conditions - A large hospital-based cross-sectional study.	Cross-sectional	28794 pregnancies	Chronic medical conditions vs. no conditions; behaviors such as folic acid intake, smoking, alcohol	Overall, ~74% planned their pregnancies; women with T2DM or mental illness were less likely to plan (aRR<1.0), but those with chronic conditions were generally more likely to adhere to recommended planning behaviors.
Schaffer et al. 1998	9710657	Energy and nutrient intakes and health practices of Latinas and white non-Latinas in the 3 months before pregnancy.	Cross-sectional	462 women (one-third Latina; 58.1% of Latinas were foreign-born)	Dietary and supplement intake, plus other health practices, in the 3 months before pregnancy	Mean/median energy intake exceeded 2,000 kcal/day in all groups, and fewer than half consumed ≥5 servings of fruits/vegetables daily. Foreign-born Latinas had the lowest fat percentage of total energy, highest carbohydrate, cholesterol, fiber, grains, protein, folate, vitamin C, iron, and zinc. Half of all women's iron intake was below the RDA.
Umer et al. 2016	27351732	Factors Associated with Receipt of Pre-pregnancy Preventive Dental Care Among Women in West Virginia: Pregnancy Risk Assessment Monitoring System (PRAMS) Survey 2009-2010.	Cross-sectional	3,050 women from the 2009–2010 West Virginia PRAMS dataset	Sociodemographic, economic, and health-related lifestyle factors associated with receiving dental cleaning prior to pregnancy	About 47% of women had a dental cleaning before pregnancy. Adjusted odds of pre-pregnancy dental cleaning were higher among Non-Hispanic white women (OR 1.75; 95% CI 1.01–3.04), those with >high school education (OR 1.79; 95% CI 1.22–2.62), private health insurance (OR 2.65; 95% CI 1.98–3.55), intended pregnancy (OR 1.30; 95% CI 1.04–1.64), and women under age 20 (OR 2.75; 95% CI 1.86–4.06).
Zhao et al. 2014	25366578	Factors influencing the quality of preconception healthcare in China: applying a preconceptional instrument to assess healthcare needs.	Cross-sectional	3,202 women of reproductive age (2,806 completed questionnaires: 1,011 from Jiangsu, 1,795 from Hebei)	Quality and content of preconception healthcare services in two regions of China (eastern vs. northern), including demographic, health status, immunization, and need for care	Significant differences by region in maternal age ($p<0.001$), BMI ($p<0.001$), education ($\chi^2=916.33$, $p<0.001$), occupation ($\chi^2=901.78$, $p<0.001$), health status/common disease, immunizations, and demand for PCC. Authors suggest modifying national guidelines regionally based on local demographics and improving public education and professional services.
Ersek et al. 2009	19883477	Physical activity prior to and during pregnancy and risk of postpartum depressive symptoms.	Cross-sectional	2,169 new mothers (18–45 y) in North Carolina, from 2004–2005 PRAMS dataset	Self-reported physical activity before pregnancy and in the last trimester, and postpartum depressive symptoms (feeling down, little interest/pleasure)	After adjustment, no significant associations between physical activity and "feeling down." However, women physically active both before and late in pregnancy showed lower odds of having "little interest/pleasure" postpartum (OR=0.66; 95% CI 0.49–0.87).

Xaverius et al. 2012	21161383	Preconception wellness: differences in health by immigrant status.	Cross-sectional	8,095 reproductive-age women (NHANES 1999–2006), comparing US-born vs. foreign-born, pregnant vs. nonpregnant	Immigration status and pregnancy status in relation to multiple health behaviors (BMI, smoking, binge drinking, illicit drug use, physical activity, birth control use)	Pregnant US-born women used illicit drugs 5.2× more than pregnant foreign-born. Nonpregnant US-born women had significantly higher rates of illicit drug use (3.7×), lower likelihood of normal BMI (–45%), 2× higher binge drinking, 7.6× higher smoking, but more moderate exercise vs. nonpregnant foreign-born. Immigrant women demonstrated healthier profiles in several lifestyle behaviors.
Paulik et al. 2009	19411131	Preconceptional and prenatal predictors of folic acid intake in Hungarian pregnant women.	Cross-sectional	349 pregnant women at a single center, questionnaire-based study	Folic acid intake timing (preconception vs. prenatal) and associated demographic, obstetric, and pregnancy care factors	Planning of pregnancy, prior infertility treatment, and use of multivitamins were linked to higher rates of preconception folic acid usage. Probability of prenatal folic acid intake rose with maternal age and earlier pregnancy detection, and was strongly related to preconception folic acid use. Authors emphasize the need for targeting women less likely to take folic acid.
Katko et al. 2018	28593684	Thyroglobulin level at week 16 of pregnancy is superior to urinary iodine concentration in revealing preconceptual and first trimester iodine supply.	Cross-sectional	164 pregnant women, cross-sectional measurement at 16 weeks gestation	Timing of iodine supplementation (≥150 μg/day) before or after conception, maternal serum thyroglobulin (Tg), urinary iodine concentration (UIC), and smoking status	Median UIC was adequate overall (162 μg/L). Women supplementing ≥150 μg/day had higher UIC than nonusers. Those starting ≥4 weeks pre-pregnancy had significantly lower serum Tg (9.1 μg/L) vs. pregnancy starters (14.5) and nonusers (14.6), indicating better iodine status. Regression showed Tg was inversely related to supplementation duration ($p=0.010$) and positively associated with smoking ($p=0.008$).
